# Full-space inverse-designed meta-optics for complex vector field shaping of intracavity landscapes

**DOI:** 10.1038/s41377-026-02258-w

**Published:** 2026-04-03

**Authors:** Mingfeng Xu, Di Sang, Mingbo Pu, Ping Gao, Bowen Zhao, Yuhan Zheng, Qingji Zeng, Lianwei Chen, Fei Zhang, Yinghui Guo, Xiong Li, Xiaoliang Ma, Yunqi Fu, Xiangang Luo

**Affiliations:** 1 State Key Laboratory of Optical Field Manipulation Science and Technology, Chinese Academy of Sciences, Chengdu, 610209 China; 2 Research Center on Vector Optical Fields, Institute of Optics and Electronics, Chinese Academy of Sciences, Chengdu, 610209 China; 3 College of Materials Science and Opto-Electronic Technology, University of Chinese Academy of Sciences, Beijing, 100049 China; 4 College of Electronic Science and Technology, National University of Defense Technology, Changsha, 410073 China

**Keywords:** Metamaterials, Nanophotonics and plasmonics

## Abstract

The sophisticated shaping of electromagnetic field landscapes, both outside and inside optical cavities, has long been an important goal of optics and photonics, with broad applications in near-field imaging, lithography, and laser science. Despite extensive progress in far-field shaping of extracavity landscapes, achieving accurate tailoring of three-dimensional intracavity fields has remained elusive. Here, we propose full-space adjoint-enabled freeform meta-optics for complex, on-demand vector field shaping of intracavity landscapes. In contrast with conventional semi-space adjoint approaches, the full-space adjoint strategy allows for efficient full-wave optimization of vector fields at the subwavelength scale, overcoming strong multiple scattering and interference intrinsic to enclosed cavity boundaries. We experimentally demonstrate tailored plasmonic cavity landscapes with a freeform metasurface mask, realizing a fivefold enhancement in imaging fidelity without sacrificing optical super-resolution performance. Our work significantly broadens the scope of freeform meta-optics and may open new avenues for applications in nanophotonics, topological photonics, and quantum optics.

## Introduction

A fundamental challenge in optical field shaping arises from the nature of near-field and far-field propagation. When an electromagnetic wave propagates from near-field to far-field, high spatial frequency components are confined within a near-field region as evanescent waves. This confinement mechanism prevents the collection of high-resolution spatial information at far-field^1^. Recently, several techniques have been proposed to shape or recover near-field landscapes using far-field methods, based on classical antenna theory^2^ or the counterpropagating guided-waves interference method^3^. Beyond those approaches for extracavity configurations, shaping the intracavity field landscapes within an optical nanocavity has also been a long-standing endeavor, offering promising applications in laser^4^, meta-optics^5,6^, topological photonics^7,8^, and quantum technology^9^. In contrast to extracavity scenarios, however, the complex interplay of multiple scattering, interference and vector field effect emerging in the intracavity environments makes shaping intracavity landscapes significantly more sophisticated. To date, no effective approach has been well developed to address these challenges.

To address these intricate design challenges, adjoint-based topology optimization has emerged as a general and computationally efficient inverse-design method in nanophotonics^10,11^. Its versatility has been demonstrated across optimization problems in linear optics^12–14^, exceptional and topological photonics^15,16^, nonlinear optics^15,17^, and even in regimes of wave chaos^18,19^. Recently, the rapid development of freeform meta-optics—inverse-designed metasurfaces with topology optimization approach—has unlocked the possibility to design freeform meta-atoms and control light with multiple degrees of freedom at subwavelength scale^20–22^, enabling large-area, high-numerical-aperture devices^23^. Complex shaping of subwavelength light field with freeform meta-optics has been widely demonstrated for a plethora of applications, including freeform metagrating^24–27^, freeform metalens^28–35^, polarization conversion metasurfaces^36–38^, near-field enhancement^39,40^, and others^41–44^. To date, freeform meta-optics has been largely restricted to extracavity implementations in either the near or far field. In contrast, sophisticated shaping of the three-dimensional intracavity field with freeform meta-optics remains elusive, primarily due to the enclosed boundaries of optical cavities that induce strong multiple scattering between forward (*k*_*z*_ > 0) and backward (*k*_*z*_ < 0) waves—a phenomenon absent in semi-open extracavity settings.

Here, we propose a general framework of freeform meta-optics that employs a full-space adjoint approach to achieve complex, on-demand vector field shaping of intracavity landscapes. Specifically, the full-space adjoint approach overcomes the complex multiple scattering and vectorial polarization challenges within an optical cavity, thereby making it possible to realize sophisticated shaping of the three-dimensional intracavity field at subwavelength scale. As a proof-of-concept demonstration, the full-space adjoint approach is applied to a typical plasmonic cavity for high-fidelity optical sub-diffractional imaging^45–57^, where two polarization-dependent full-space adjoint simulations involving full vector components of the electric field are explored to calculate the gradient information of the freeform metasurface mask. According to the symmetry of Green’s function, this strategy enables efficient performance of full-wave optimization of complex vector fields at the subwavelength scale. Consequently, the near-field imaging fidelity of the plasmonic cavity is improved, even with limited spectral information. The experimental results show that the optimized patterns achieve a notably higher imaging fidelity, particularly for a variety of complex images with subwavelength feature size. Specifically, the average area error ratio (AER) of the locally optimized patterns is greatly improved by fivefold, with an optical resolution of ~ *λ*/5, where *λ* denotes the incident wavelength.

## Results

### Concept and challenges of complex shaping of intracavity landscapes

**Fig. 1:**
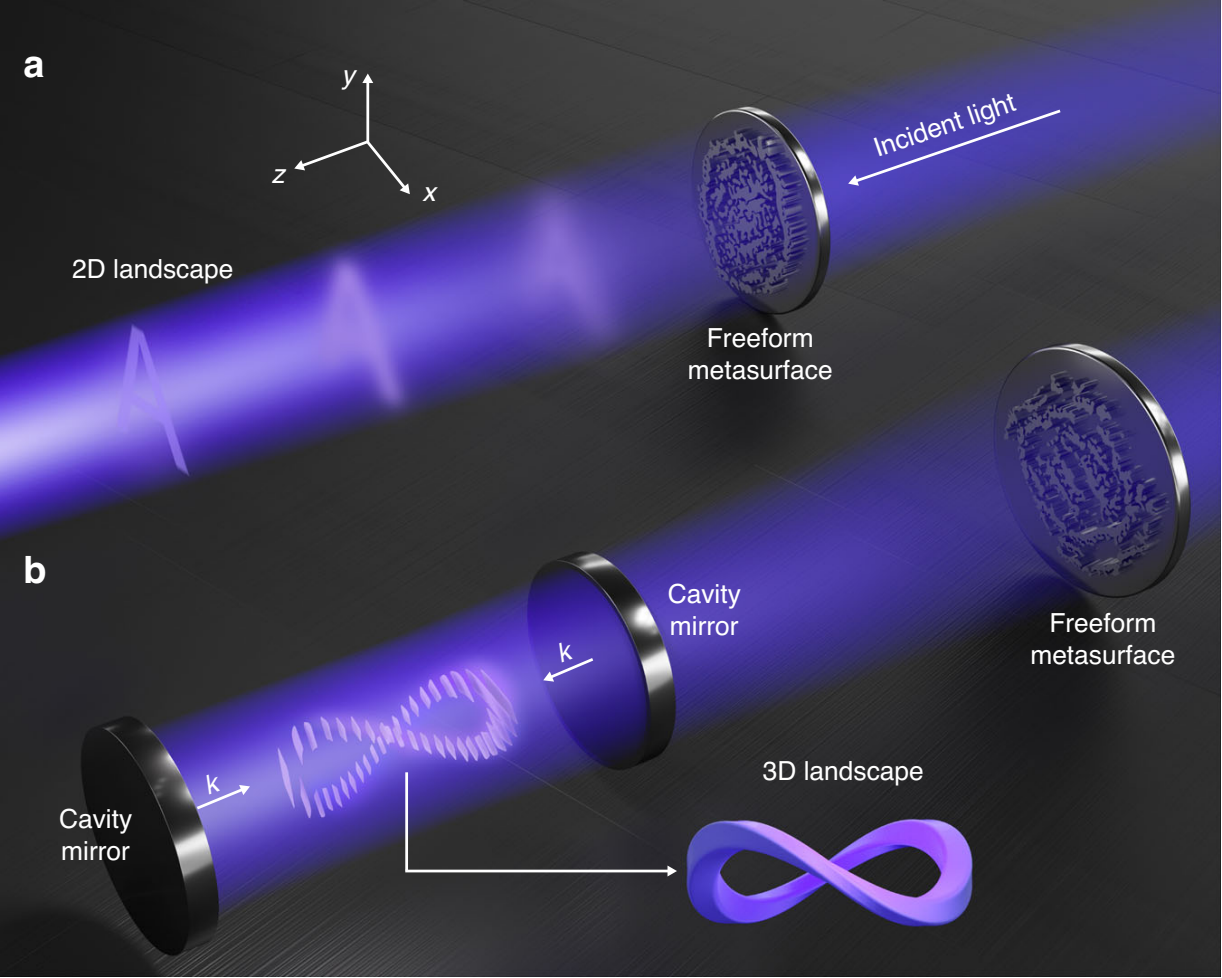
**Schematics of complex shaping with meta-optics.** **a** For extracavity, the complex shaping problem of two-dimensional landscapes corresponds to the wavefront shaping. **b** For intracavity, the complex shaping problem of three-dimensional landscapes corresponds to the vector field shaping

**Fig. 2:**
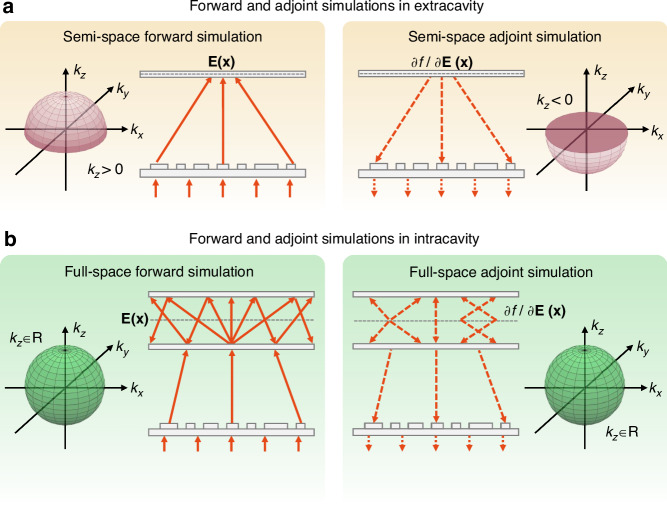
**Forward and adjoint simulations in extracavity and intracavity.** **a** For extracavity, the adjoint simulation is performed within semi-space, where the vertical wave vector *k*_*z*_ > 0 in the forward simulation and *k*_*z*_ < 0 in the adjoint simulation. **b** For intracavity, the adjoint simulation is performed in full-space, where the vertical wave vector *k*_*z*_ ∈ R in both the forward and adjoint simulations

We begin by discussing the challenges and differences in the complex shaping of electromagnetic fields both within and outside the cavity. As shown in Fig. 1a, electromagnetic waves propagating within the semi-enclosed boundary outside the cavity—comprising both near-field and far-field landscapes—can be described by the free-space diffraction equation. The complex shaping of extracavity landscapes is akin to the well-established wavefront shaping technique, which is implemented on the scalar component *E*_*x*_ of the electromagnetic field. Notably, freeform meta-optics could achieve the complex shaping of either near-field or far-field extracavity landscape by optimizing the wavefront throughout the semi-enclosed space (Fig. 2a).

In contrast, as illustrated in Fig. 1b, shaping three-dimensional landscapes at different distances inside the cavity becomes more complex due to the intrinsic characteristics of the fully enclosed boundary, which results in intricate interactions of multiple scattering and interference effects. In this scenario, the incident wave vector enters the intracavity region from one side, interacts with the opposing boundary, and generates multiple backward propagation modes that interfere with the incident field. Essentially, shaping the electromagnetic landscape inside the cavity corresponds to a vector field shaping problem, where all vector components of the electromagnetic field must be optimized simultaneously with a well-defined FoM:

1 $${\mathrm{FoM}}=\int \parallel \chi ({{\bf{E}}}_{d}{{\bf{(x)}}})-\chi ({\bf{E(x)}}){\parallel }_{2}^{2}{\mathrm{d}}{\bf{x}}$$

where **E**(***x***) indicates the actually shaped electric field vector in the cavity, **E**_*d*(***x***)_ denotes the desired electric field, and *χ*( ⋅ ) represents the projection function of electric field, specific to different shaping tasks. According to the principles of Fourier optics, any light field distribution inside the cavity can be synthesized through the linear superposition of appropriate spatial frequency components. Specifically, light waves propagating in different directions correspond to plane waves with different spatial frequencies. By modulating and superposing the amplitude and phase of these plane waves, any desired complex field distribution can be constructed. The electric field inside the cavity can be expressed as the superposition of forward and backward interference of different transverse components:

2 $${E}_{i}({\bf{x}})=\int {E}_{i}({k}_{x}){\mathrm{d}}{k}_{x}=\int ({a}_{i}{e}^{j{k}_{z}z}+{b}_{i}{e}^{-j{k}_{z}z}){e}^{j{k}_{x}x}{\mathrm{d}}{k}_{x}$$

where *i* ∈ {*x*, *y*, *z*} represents the vector field components, *a* and *b* represent the field coefficients, and *k*_*x*(*z*)_ is the component of the wave vector *k* (*k* = 2*π*/*λ*) in the *x*(*z*) direction. This process can be seen as reconstructing the optical field distribution in the spatial domain through the synthesis of the spatial spectrum. Notably, evanescent waves carrying high spatial resolution features play a crucial role in forming subwavelength complex landscapes.

### Principle of full-space adjoint in an optical cavity

To achieve the shaping of complex vector fields within optical cavities, topology optimization based on adjoint simulations is a highly flexible and effective method (see Supplementary Section S1 and Materials and Methods). In this method, the forward field inside the device is obtained by exciting it with the original source, while the adjoint field is computed by placing an adjoint source at the target region. For reciprocal media, the symmetry of the Green’s function ensures that the gradient calculated via the adjoint method is valid (Eq. S7). This symmetry allows us to compute the gradient for all design variables (the permittivity distribution) through just two sets of simulations (one forward and one adjoint) per optimization iteration, rather than requiring a perturbational simulation for each variable, making full-wave vector optimization at the subwavelength scale computationally feasible. However, as shown in Fig. 2, the adjoint simulation methods are different for extracavity and intracavity scenarios, particularly in terms of boundary conditions and wave vector directions. For the extracavity with a semi-enclosed boundary (Fig. 2a), the adjoint field excited inside the device by the forward-propagating adjoint source can be considered negligible. In this case, the wave vector in the forward simulation (*k*_*z*_ > 0) is opposite to the wave vector in the adjoint simulation (*k*_*z*_ < 0). This implies that the wavefront propagation in forward and adjoint simulations only involves half-space wave vectors, thus avoiding the complexity of forward and backward propagation interference effects. In contrast, as shown in Fig. 2b, the enclosed boundary condition of the intracavity results in multiple scattering effects, thus greatly increasing the complexity of internal landscapes. In the forward simulations, full-space wave fields (*k*_*z*_ ∈ **R**) are present inside the cavity. Therefore, the adjoint sources must be added separately along the positive z (*k*_*z*_ > 0) and negative z (*k*_*z*_ < 0) directions during the adjoint simulation process. This full-space adjoint strategy provides a comprehensive and effective solution for vector field shaping of complex landscapes within different optical cavities.

### Complex vector field shaping in a plasmonic cavity

**Fig. 3:**
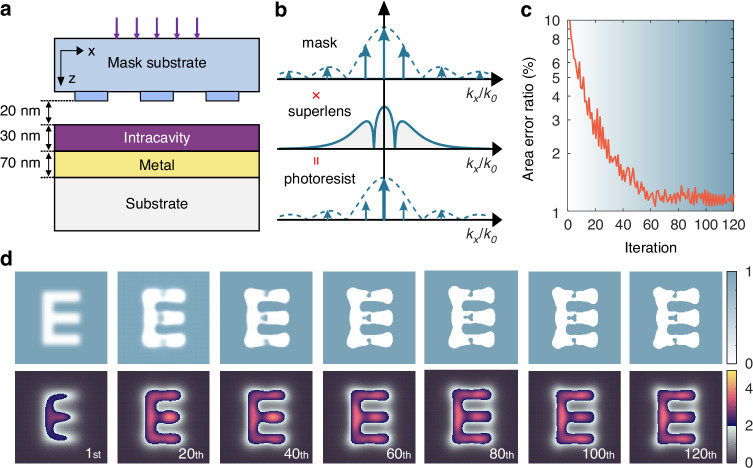
**Schematics and the plasmonic reflective superlens near-field imaging system.** **a** A typical plasmonic reflective superlens system includes a metasurface mask with subwavelength patterns on a transparent substrate, an air spacer, a photoresist film, and a metal reflective film. The intracavity region refers to the photoresist layer. **b** Spectrum product of metasurface mask and superlens results in the spectrum of the image in the photoresist in the linear imaging model. **c** Convergence process of the area error ratio during the optimization iterations. **d** Evolution process of optimized Cr metasurface mask morphology and near-field imaging patterns during the optimization iterations. Normalized intensity below the exposure threshold is shown in “gray'', while the “inferno” colormap highlights the field distribution in the “E” pattern region above the exposure threshold

As a proof-of-concept, we adopted a plasmonic cavity to demonstrate the proposed shaping approach. Herein, we define the optical cavity as the region bounded by interfaces where complex multiple scattering occurs, rather than a physically hollow enclosure. As illustrated in Fig. 3a, our system is a multilayer stack functioning as a plasmonic reflective superlens, comprising a freeform metasurface mask (Cr nanoslits) on a substrate, an air spacer, a photoresist film, and a metal reflective film. The metasurface mask itself constitutes the top boundary outside of this enclosed cavity system. The photoresist layer, where the intracavity landscape is shaped, is sandwiched inside and serves as the core region for field manipulation. Herein, the optical cavity is defined as the photoresist layer, bounded by the air/resist and resist/metal interfaces. The freeform metasurface mask serves as an external patterned illuminator to this cavity. This configuration leverages the fact that metallic materials exhibiting a negative refractive index in the optical region can excite surface plasmon polaritons (SPP)^58,59^, enabling excellent evanescent wave amplification and transmission of high-frequency spatial information^60,61^ (see section S2). However, the commonly adopted Hopkins model^62^ for conventional imaging analysis, which relies on the linear transfer matrix method (TMM) approach^63,64^ (see section S3), suffers from large aberrations in near-field imaging, where vector field effect, non-uniform evanescent waves amplification, and strong near-field coupling effect are significant. Moreover, the spectral transfer performance of the superlens is uneven due to diffraction limitations, which prevents the spectral information of the mask layer from being proportionally transferred into the photoresist cavity thin film (Fig. 3b). This results in serious pattern distortion (see section S4).

In order to improve the overall image fidelity while keeping the resolution unimpaired, full-vectorial correction and compensation of the strongly coupled near-field evanescent wave are required. Here, we adopt the aforementioned full-space adjoint-based topology optimization method as an effective tool for modulating evanescent waves of different spatial frequencies. Despite the presence of limited and uneven low spectral components, this approach can accurately reconstruct near-field information. The desired imaging pattern with arbitrary slit orientations can be achieved by superimposing the intensity distribution of non-coherent polarization-independent illumination (both TM and TE polarizations). Notably, only TM waves can excite the SPP. Moreover, for photosensitive imaging, all electric field vector components (including ∣*E*_TM,*x*_∣^2^,∣*E*_TM,*y*_∣^2^,∣*E*_TM,*z*_∣^2^) must be considered due to the complex near-field coupling effects. For the plasmonic near-field imaging system, the FoM is defined as the difference between the desired pattern *P*_*d*_ and the actual pattern *P*_*a*_. To model the photoresist development process that binarizes the continuous light intensity into a final pattern, the actual pattern *P*_*a*_ is approximated by a smooth threshold sigmoid function as the projection function applied to the total light intensity in the photoresist^65^

3 $${P}_{a}({\bf{E(x)}})\simeq {\mathrm{Sig}}\{| {{\bf{E}}}_{m}{| }^{2}-{I}_{th}\}=\frac{1}{1+\exp [-A(| {{\bf{E}}}_{TM}{| }^{2}+| {{\bf{E}}}_{{\mathrm{TE}}}{| }^{2}-{I}_{th})]}$$

where *A* represents the steepness of the curve, and *I*_*th*_ represents the threshold of the photoresist. Then, the FOM can be expressed as

4 $$FoM\simeq \int | | Sig({{\bf{E}}}_d{{\bf{(x)}}})-Sig({\bf{E(x)}})| {| }_{2}^{2}d{\bf{x}}$$

The normalized FoM can be defined as AER, which is expressed as the ratio of the mismatch region between the actual image and the target image relative to the entire area of the metal metasurface mask. The FoM for optimization was defined at the central plane of the photoresist layer, where the field intensity is maximal. This approach is well-justified as the photoresist development is a threshold process, and patterning fidelity is predominantly determined by the exposure dose at this crucial plane. A full 3D optimization across the entire photoresist volume was deemed computationally intractable and unnecessary for achieving high-fidelity pattern transfer.

Due to the double-polarization exposure, each iteration of optimization requires four steps (see Fig. S12): forward and adjoint simulations for both TM and TE wave excitations. Thus, the two adjoint sources are expressed as a superposition of three vector-field components (see section S5)

5 $$\left\{\begin{array}{l}2A({P}_{a}-{P}_{d}){{P}_{a}}^{2}\exp [-A(| {{\bf{E}}}_{\mathrm{TM}}{| }^{2}+| {{\bf{E}}}_{\mathrm{TE}}{| }^{2}-{I}_{th})]{{\bf{E}}}_{\mathrm{TM}}^{* },\,\mathrm{for}\,\mathrm{TM}\,\mathrm{adjoint}\\ 2A({P}_{a}-{P}_{d}){{P}_{a}}^{2}\exp [-A(| {{\bf{E}}}_{\mathrm{TM}}{| }^{2}+| {{\bf{E}}}_{\mathrm{TE}}{| }^{2}-{I}_{th})]{{\bf{E}}}_{\mathrm{TE}}^{* },\,\mathrm{for}\,\mathrm{TE}\,\mathrm{adjoint}\end{array}\right.$$

The vector field optimization based on adjoint algorithm enables the structural variation of the freeform metasurface mask, as well as fast convergence without additional interventions. Figure 3c illustrates the convergence of the FoM during the optimization process, showing a rapid decrease in AER within the first 60 iterations, after which the curve stabilizes. Figure 3d represents the topological evolution and binarization process of the metasurface mask, as well as the corresponding correction process of the imaging pattern. The most significant changes in the optimized metasurface masks occur during the first 60 iterations, accompanied by a great improvement in the imaging fidelity. The latter 60 iterations focus primarily on fine corrections, gradually achieving full mask binarization. The optimized mask exhibits clear topological changes, such as the emergence of new metal posts in originally empty regions. These simulation results clearly show the strong capability of the adjoint-based optimization method to utilize the limited spatial information for the fidelity enhancement of near-field imaging. Note that the image quality of topology-optimized mask exhibits considerable robustness under practical environment perturbation (such as angular misalignments in the cavity assembly) and fabrication errors (see sections S6 and S7). Moreover, a further and comparative analysis confirms that, compared to the semi-space adjoint method, the full-space adjoint formulation is essential to obtain high-fidelity solutions for complex field shaping of enclosed cavities where coherent multi-scattering is intrinsic (see section S8 for more details).

### Experimental demonstration

**Fig. 4:**
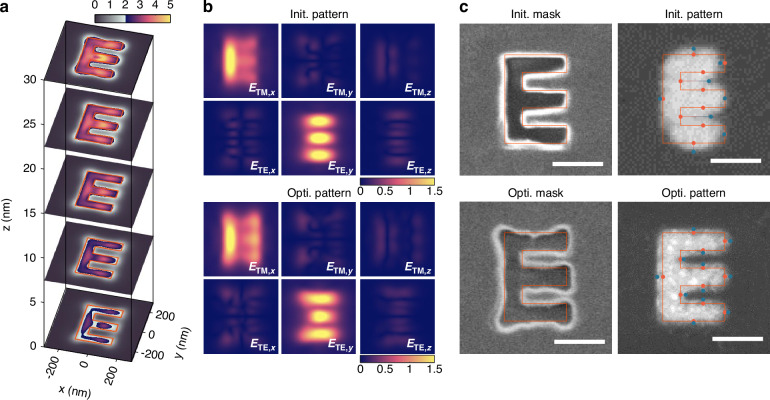
**Locally optimized results of the image “E” in the plasmonic near-field imaging system.** **a** Cross-section view of the simulated normalized light field distribution in different regions of the photoresist film. **b** Full vector field analysis of initial and optimized imaging pattern at the central cross-section (*z* = 15 nm) of the photoresist film. **c** Scanning electron microscopy of the metasurface mask and imaging results. The red frames indicate the target pattern. Red and blue points mark the corresponding target and imaged edge locations, respectively; their separation defines the EPE. Scale bar: 200 nm

We then experimentally demonstrate the fidelity enhancement for a single image using the locally optimal tailoring method. Figure 4a indicates the simulated 3D intensity distribution of a locally optimized image “E”, which is distributed at 7.5 nm intervals inside the photoresist film. In comparison with the results based on TMM and finite difference time-domain (FDTD) methods (Fig. S4c in section S4), the corresponding imaging fidelity is significantly improved. Specifically, at the central cross section (*z* = 15 nm), the intensity distributions within the specified “E” region are above the exposure threshold, exhibiting a remarkable E-shaped profile. In fact, such improvement stems from the combined effect of the three-dimensional vector fields under the polarization-dependent illumination (Fig. 4b). For example, the TM-polarized wave mainly excites the *x*-component of the imaging field and contributes to the lines in the *y*-direction, while the TE-polarized wave mainly excites the *y*-component of the imaging field and contributes to the lines in the *x*-direction. Note that the longitudinal vector field components (*E*_TM,*z*_ and *E*_TE,*z*_) also exhibit a similar distribution pattern to the main component, thereby improving edge-profile fidelity. In contrast to the initial imaging pattern, the optimized pattern has a more significant distribution of *E*_TM,*x*_ and *E*_TE,*y*_, thus leading to the fidelity enhancement of near-field imaging.

Figure 4c presents the experimental results of plasmonic near-field imaging (see the Method and Fig. S12 for the metasurface mask fabrication and the near-field imaging procedure). For the initial pattern, there are two categories of local imaging anomalies: inward shrinkage anomalies (where the photoresist contour lies inside the target contour) and outward extension anomalies (where the photoresist contour lies outside the target contour). Those anomalies lead to the inevitable distortions with the AER of 5.83% in the initial imaging pattern, especially for the corners and vertices of “E” region. In contrast, the corner in the optimized metasurface mask is expanded to compensate for the shrinkage distortion, while the corresponding center of the long edge is tightened to suppress the extension of the strong focus. As a result, the optimized imaging pattern achieves almost full coverage in the specified region and leads to a much lower AER of 1.23%, corresponding to a 4.7-fold improvement in fidelity. In addition, the local imaging quality is markedly enhanced, as reflected by the reduction of the maximum Edge Placement Error (EPE) from 111 nm to 22 nm, and the average edge error from 35 nm to 16 nm.

**Fig. 5:**
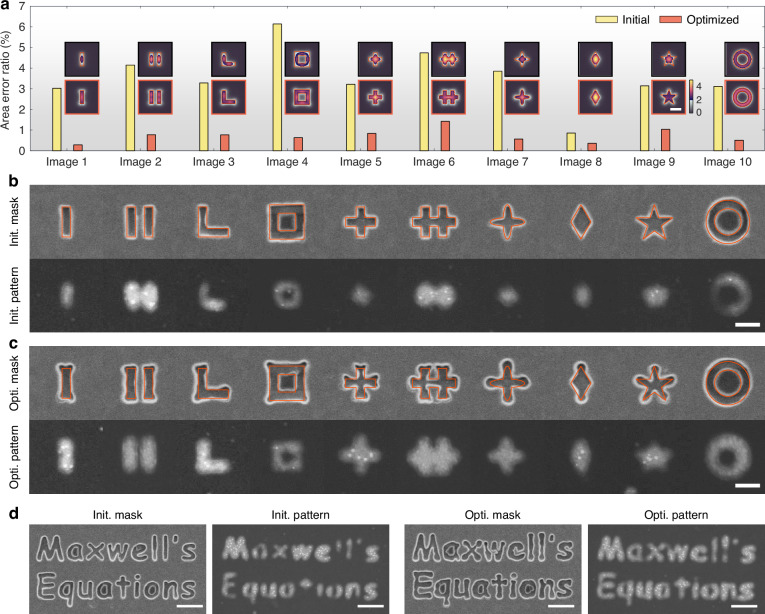
**Experimental optimization results for different types of images.** **a** Simulation results of AER before and after local optimization for 10 different single images. **b** Experimental results of initial metasurface masks and initial imaging patterns. **c** Experimental results of optimized metasurface masks and optimized imaging patterns, where the red frames indicate the target patterns. **d** Initial metasurface mask, optimized metasurface mask, and their corresponding imaging results by scanning electron microscopy for “Maxwell’s Equations” image. The image sizes are 600 nm × 600 nm in (**a**–**c**) and 3.4 μm × 1.7 μm in (**d**), both with the minimum feature width of 70 nm. Scale bar: (**a**–**c**) 200 nm, (**d**) 500 nm

To certify the general capability of the locally optimal tailoring method, the fidelity improvements for different images are investigated. Figure 5a presents the simulation results of the AER for the other ten different images, ranging from single-line and cross to pentagram and circular ring. Convergence analyses for all geometric patterns are provided in Supplementary Section S9. Compared to initial patterns, the average AERs of locally optimized patterns are greatly reduced from 3.5% to 0.7%. In particular, the optimization result of a single line (image 1) shows a maximum improvement of ~10 times in fidelity. Experimental results agree well with the simulation results, as shown in Fig. 5b, c. For instance, the right-angle line in the optimized metasurface mask has a more curved outline with a protruding boundary at the corner, correcting near-field imaging errors by selectively enlarging the transmission area of the metasurface mask with high spatial frequency components. The variation in fidelity improvement stems from pattern complexity. Simple patterns like a single line exhibit stronger enhancement because they contain fewer spatial frequencies, experience less near-field coupling, and are more sensitive to a single polarization component, making them more amenable to optimization.

Finally, the fidelity enhancement performance is demonstrated for large-area images. Figure 5d presents the experimental results of a complex imaging pattern of “Maxwell’s equations” with a size of 3.4 μm × 1.7 μm. Compared to the initial pattern, the optimized image shows a significant improvement in near-field imaging fidelity. Missing parts in the initial pattern, such as “l” and “t” are recovered through local optimization, reducing the AER from 2.336% to 0.998% (see Fig. S14). However, it should be noted that the pattern “e” appears as a pattern “c” due to the approximately 40 nm feature size, which exceeds the system’s imaging resolution. Additionally, our method demonstrates robust fidelity improvement across various scales, from large-area images with 70 nm features to periodic patterns with 50 nm linewidths (see section S10 and S11). Resolution tests confirm that the system reliably resolves features down to 70 nm ( ~ *λ*/5) with high fidelity. Features beyond this limit (e.g., 50 − 60 nm) suffer from intrinsic evanescent decay and are not consistently resolved. Notably, the optimization process enhances pattern fidelity—particularly near the resolution limit—but may suppress weak high-frequency components corresponding to sub-resolution features, occasionally rendering them less discernible than in the initial design. This fidelity-resolution trade-off arises because the inverse-design prioritizes field matching without explicitly enforcing geometric simplicity, sometimes leading to more complex mask patterns (Fig. S10). Fabrication constraints further accentuate these structural discontinuities.

## Discussion

Our work establishes a general inverse-design framework for meta-optics operating within enclosed cavities, a domain distinct from the conventional free-space design paradigm. The key innovation lies in our full-space vectorial adjoint method. This fundamentally diverges from the semi-space scalar approaches used in state-of-the-art large-scale metasurface design. References^31^ and^66^ achieve remarkable performance in free space by innovating in forward simulation (using a fast Chebyshev surrogate model) and gradient calculation (employing a local optimization or Fourier-based filtering). However, both works retain the traditional semi-space scalar adjoint simulation, which only considers outgoing waves (*k*_*z*_ > 0) and a limited set of field components (∣*E*_*x*_∣^2^, ∣*E*_*y*_∣^2^). In contrast, our method is specifically engineered for the intracavity regime: it computes the adjoint field in full-space (${k}_{z}\in {\mathbb{R}}$) to account for backwards-propagating modes and employs a vectorial formulation that simultaneously optimizes all electric field components (e.g., ∣*E*_TM,*x*_∣^2^, ∣*E*_TM,*y*_∣^2^, ∣*E*_TM,*z*_∣^2^) to mitigate complex near-field coupling. This capability, demonstrated here for near-field imaging, opens new avenues for tailoring light-matter interactions in cavities for quantum emission, nanolasing, and strong coupling. While the present demonstration employs a 2.5D geometry for efficiency, the full-space adjoint formalism is inherently three-dimensional and can be directly extended to shape arbitrary 3D vector landscapes by optimizing volumetric meta-structures, with the computational cost scaling with the design domain size.

Our work demonstrates that the proposed full-space adjoint-based freeform meta-optics is an efficient approach to achieving complex shaping of intracavity landscapes. In comparison with traditional gradient-based optimization techniques^67–70^, it has high computational efficiency as well as large design degrees of freedom. In the case of a plasmonic reflective cavity, this approach enables iterative updates to the freeform metasurface mask design, allowing for locally optimized tailoring of full-vectorial evanescent waves. This approach results in a significant enhancement of optical near-field imaging fidelity, without sacrificing the performance of optical super-resolution. Importantly, further refinement of image fidelity could be realized by combining our work with other traditional proximity-effect correction methods, such as off-axis illumination and peripheral grooves^56^. In the future, to steer designs towards more manufacturable geometries, one could incorporate additional regularization terms (e.g., penalizing perimeter length or enforcing minimum feature size) directly into the FoM, or apply post-processing filters constrained by the lithography process. Here, we used a simple Gaussian filter (radius 20 nm) and a gradual binarization projection to ensure feature sizes were above our fabrication limit (~20–30 nm), but more advanced constraint handling could further improve robustness.

Beyond superlens imaging, our method can be extended to other intracavity applications such as quantum emitter coupling, topological photonic cavities, and nonlinear frequency conversion. The full-space adjoint framework provides a universal tool for complex vector field engineering in enclosed photonic systems. Beyond near-field imaging and lithography, the proposed full-space inverse-design framework is generically applicable to a wide range of intracavity field engineering problems. Potential applications include enhancing light-matter interactions in quantum optical cavities for single-photon source efficiency, controlling the emission patterns of nanoscale lasers, engineering band structures in topological photonic crystals, and achieving arbitrary vector field shaping for advanced sensing platforms.

In addition to the freeform metasurface mask optimization, the proposed approach can be generically extended to other scenarios in super-resolution imaging and lithography. For example, by inversely optimizing the phase and/or the vector-polarization state of illumination light using a full-space adjoint-based topology optimization method, one can further excite high spatial frequency information involved in the near-field imaging process. On the other hand, the presented high-fidelity and super-resolution imaging capability also has a wide range of applications in nanophotonics and metasurfaces. For example, it could be directly used to fabricate the freeform periodic subwavelength array as a Fano-resonant metasurface (see section S11), which has promising applications in sensing and optical modulation^71^. Moreover, further enhancements in complex shaping can be achieved by integrating deep learning approaches^72–74^ and multifunctional polarization multiplexing techniques^75,76^. Future enhancements could explore neural reparameterization to implicitly enforce manufacturing constraints and improve feature size control during optimization^77^. Our results may inspire a wide variety of new subwavelength cavity-related meta-devices with exciting applications in meta-optics, nanophotonics, and quantum optics.

## Materials and methods

### Full-mode simulation of plasmonic cavity

The FDTD method was used to analyze the electric field intensity distributions in a plasmonic cavity. All simulations were performed on a computer with an Intel Core i5-6300HQ CPU, 2.3 GHz, and 64 GB of RAM. In the simulation, the thicknesses of the air gap, photoresist and Ag were set to 20 nm, 30 nm, and 70 nm, respectively. The light source was set to a plane wave with a wavelength of 365 nm. Moreover, the complex permittivities of Cr, Ag and Si were set to as *ε*_Cr_ = − 8.55 + 8.96i, *ε*_Ag_ = − 2.17 + 0.36i, *ε*_Si_ = 35.71 + 35i, respectively. The permittivity of SiO_2_ and photoresist were set to 2.17 and 2.59, respectively. For the thin (30 nm) photoresist used here, the field variation along the cavity axis (*z*) is relatively uniform (see Fig. S4). The FoM was therefore defined at the central plane (*z* = 15 nm), which is representative of the exposure dose throughout the resist thickness and sufficient for high-fidelity patterning in our system.

### Topology optimization simulation

The pixel size of the topology-optimized structure was chosen to be 1 nm to provide sufficient design freedom. The initial structure in the iterative process was a informationally ambiguous target pattern. Then, the metasurface mask was iteratively updated based on the three field-component values derived from the four simulations (two forward simulations and two adjoint simulations). The two forward simulations were independently excited by TM and TE-polarized plane waves, respectively, while the two adjoint simulations were independently excited by the adjoint sources, as indicated in Eq. (5). Moreover, a density filter with a radius of 20 nm was applied in each iteration to enforce a minimum length scale and ensure manufacturability. To convert the continuous density field $\widetilde{\rho }({\bf{x}})$ (varying between 0 for air and 1 for Cr) to a binary mask, a smoothed Heaviside projection function $\overline{\rho }=\frac{\tanh (\beta \eta )+\tanh (\beta (\widetilde{\rho }-\eta ))}{\tanh (\beta \eta )+\tanh (\beta (1-\eta ))}$ was used, where *η* = 0.5 is the threshold and *β* controls the sharpness. *β* was gradually increased from 1 to 64 over the 120 iterations to progressively push the densities towards 0 or 1, thus ensuring final binarization. Recent methods, such as subpixel-smoothed projection, can alleviate the convergence difficulties associated with this *β*-continuation process^78^. No additional penalty terms were used. This enforced binarization is crucial as it ensures the final design corresponds to a physically realizable Cr/air mask. While it may slightly constrain the theoretical FoM compared to a greyscale structure, it is essential for experimental validation. The effective complex permittivity at each pixel was interpolated between air and chromium using the filtered and projected density $\overline{\rho }$: $\varepsilon (\overline{\rho })={\varepsilon }_{air}+\overline{\rho }({\varepsilon }_{Cr}-{\varepsilon }_{air})$. The filtering and projection are applied to the scalar density field, ensuring a physically consistent transition during optimization. The shape parameter of the projection function was gradually increased throughout the iterations, ensuring that the final pattern was fully binarized. Finally, the symmetric and anti-symmetric boundary conditions were added according to the symmetries of both the structure and source, accelerating the optimization process. The computational cost for one optimization iteration (involving two forward and two adjoint FDTD simulations) was ~1 minute on our workstation. The full optimization for a pattern like the “E” (120 iterations) required ~2 hours.

### Fabrication of Cr metasurface mask

A 60 nm Cr film as the spacer and pattern film was firstly sputtered on a pre-cleaned fused silica substrate by O_2_ plasma (see Fig. S13a). To control the working distance between the mask patterns and the photoresists in the final SPP super-resolution near-field imaging, a part of the Cr film was thinned by 20 nm using focused ion beam. Then, the thinned 40 nm Cr film was patterned by a standard electron beam lithography procedure, and the ion beam etching technique was utilized to transfer the designed pattern to the 40 nm Cr film.

### SPP super-resolution near-field imaging procedure

A 70 nm-thick Ag film was deposited on a Si wafer via thermal evaporation at a rate of 5 nm/s under a pressure of 5.0 × 10^−4^ Pa (see Fig. S13b). Due to the mirroring effect in our face-down imaging setup, the mask pattern was pre-inverted accordingly. For clarity, all mask layouts in the figures are presented in their intended (non-inverted) orientation. A 30 nm layer of positive-tone photoresist (XT 8710) was then spin-coated onto the Ag film and hard-baked at 100 °C for 5 min. The photoresist was brought into physical contact with the Cr metasurface mask under ~0.5 MPa pressure. Exposure was carried out using a 365 nm mercury lamp (spectral range 365 nm ± 10 nm) with a divergence angle of ± 3° and a uniform flux of 4.2 mW/cm^2^. The exposure time was optimized between 10 and 20 seconds. Polarization was controlled using a linear polarizer. After exposure, the sample was developed in a 1:1 diluted AR 300-35 (All-Resist GmbH, Strausberg) solution for 40 seconds. Prior to scanning electron microscopy (SEM) imaging (Hitachi S-8010), a few-nanometer-thick Au layer was deposited to mitigate charging effects.

## Supplementary information

Supporting Information for Full-space inverse-designed meta-optics for complex vector field shaping of intracavity landscapes.

## Data Availability

All the data used to generate the plots and support the findings reported in this study are available from the corresponding authors upon request.

## References

[1] Cheben, P. et al. Subwavelength integrated photonics. *Nature* **560**, 565–572 (2018). 30158604 10.1038/s41586-018-0421-7

[2] Wei, S. B. et al. Toward broadband, dynamic structuring of a complex plasmonic field. *Sci. Adv.* **4**, eaao0533 (2018). 29868639 10.1126/sciadv.aao0533 PMC5983914

[3] Ginis, V. et al. Remote structuring of near-field landscapes. *Science* **369**, 436–440 (2020). 32703876 10.1126/science.abb6406

[4] Wu, S. F. et al. Monolayer semiconductor nanocavity lasers with ultralow thresholds. *Nature* **520**, 69–72 (2015). 25778703 10.1038/nature14290

[5] Shaltout, A. M. et al. Ultrathin and multicolour optical cavities with embedded metasurfaces. *Nat. Commun.* **9**, 2673 (2018). 29991722 10.1038/s41467-018-05034-6 PMC6039493

[6] Baßler, N. S. et al. Metasurface-based hybrid optical cavities for chiral sensing. *Phys. Rev. Lett.* **132**, 043602 (2024). 38335329 10.1103/PhysRevLett.132.043602

[7] Ozawa, T. et al. Topological photonics. *Rev. Mod. Phys.* **91**, 015006 (2019).

[8] Smirnova, D. et al. Nonlinear topological photonics. *Appl. Phys. Rev.* **7**, 021306 (2020).

[9] Lodahl, P., Mahmoodian, S. & Stobbe, S. Interfacing single photons and single quantum dots with photonic nanostructures. *Rev. Mod. Phys.* **87**, 347–400 (2015).

[10] Jensen, J. S. & Sigmund, O. Topology optimization for nano-photonics. *Laser Photonics Rev.* **5**, 308–321 (2011).

[11] Molesky, S. et al. Inverse design in nanophotonics. *Nat. Photonics* **12**, 659–670 (2018).

[12] Jensen, J. S. & Sigmund, O. Systematic design of photonic crystal structures using topology optimization: low-loss waveguide bends. *Appl. Phys. Lett.* **84**, 2022–2024 (2004).

[13] Riishede, J. & Sigmund, O. Inverse design of dispersion compensating optical fiber using topology optimization. *J. Opt. Soc. Am. B* **25**, 88–97 (2008).

[14] Yang, K. Y. et al. Inverse-designed non-reciprocal pulse router for chip-based LiDAR. *Nat. Photonics* **14**, 369–374 (2020).

[15] Lin, Z. N. et al. Cavity-enhanced second-harmonic generation via nonlinear-overlap optimization. *Optica* **3**, 233–238 (2016).

[16] Christiansen, R. E., Wang, F. W. & Sigmund, O. Topological insulators by topology optimization. *Phys. Rev. Lett.* **122**, 234502 (2019). 31298901 10.1103/PhysRevLett.122.234502

[17] Sitawarin, C. et al. Inverse-designed photonic fibers and metasurfaces for nonlinear frequency conversion [Invited]. *Photonics Res*. **6**, B82-B89 (2018).

[18] Wang, Q. Q. Forward and adjoint sensitivity computation of chaotic dynamical systems. *J. Comput. Phys.* **235**, 1–13 (2013).

[19] Blonigan, P. J. & Wang, Q. Q. Least squares shadowing sensitivity analysis of a modified Kuramoto-Sivashinsky equation. *Chaos Solit. Fractals* **64**, 16–25 (2014).

[20] Elsawy, M. M. R. et al. Numerical optimization methods for metasurfaces. *Laser Photonics Rev.* **14**, 1900445 (2020).

[21] Li, Z. Y. et al. Empowering metasurfaces with inverse design: Principles and applications. *ACS Photonics* **9**, 2178–2192 (2022).

[22] Ji, W. Y. et al. Recent advances in metasurface design and quantum optics applications with machine learning, physics-informed neural networks, and topology optimization methods. *Light Sci. Appl.* **12**, 169 (2023). 37419910 10.1038/s41377-023-01218-y PMC10328958

[23] Zhou, Y. et al. Large-area, high-numerical-aperture, freeform metasurfaces. *Laser Photonics Rev.* **18**, 2300988 (2024).

[24] Sell, D. et al. Large-angle, multifunctional metagratings based on freeform multimode geometries. *Nano Lett.* **17**, 3752–3757 (2017). 28459583 10.1021/acs.nanolett.7b01082

[25] Phan, T. et al. High-efficiency, large-area, topology-optimized metasurfaces. *Light Sci. Appl.* **8**, 48 (2019). 31149333 10.1038/s41377-019-0159-5 PMC6538635

[26] Xu, M. F. et al. Topology-optimized catenary-like metasurface for wide-angle and high-efficiency deflection: from a discrete to continuous geometric phase. *Opt. Express* **29**, 10181–10191 (2021). 33820151 10.1364/OE.422112

[27] Zhou, Y. et al. Multiresonant nonlocal metasurfaces. *Nano Lett.* **23**, 6768–6775 (2023). 37307588 10.1021/acs.nanolett.3c00772

[28] Lin, Z. N. et al. Topology optimization of freeform large-area metasurfaces. *Opt. Express* **27**, 15765–15775 (2019). 31163767 10.1364/OE.27.015765

[29] Chung, H. & Miller, O. D. High-NA achromatic metalenses by inverse design. *Opt. Express* **28**, 6945–6965 (2020). 32225932 10.1364/OE.385440

[30] Mansouree, M. et al. Large-scale parametrized metasurface design using adjoint optimization. *ACS Photonics* **8**, 455–463 (2021).

[31] Li, Z. Y. et al. Inverse design enables large-scale high-performance meta-optics reshaping virtual reality. *Nat. Commun.* **13**, 2409 (2022). 35504864 10.1038/s41467-022-29973-3 PMC9064995

[32] Sang, D. et al. Toward high-efficiency ultrahigh numerical aperture freeform metalens: From vector diffraction theory to topology optimization. *Laser Photonics Rev.* **16**, 2200265 (2022).

[33] Zheng, Y. H. et al. Designing high-efficiency extended depth-of-focus metalens via topology-shape optimization. *Nanophotonics* **11**, 2967–2975 (2022). 39634086 10.1515/nanoph-2022-0183 PMC11501133

[34] Ha, Y. L. et al. Physics-data-driven intelligent optimization for large-aperture metalenses. *Opto-Electron. Adv.* **6**, 230133 (2023).

[35] Li, S. Y., Lin, H. C. & Hsu, C. W. High-efficiency high-numerical-aperture metalens designed by maximizing the efficiency limit. *Optica* **11**, 454–459 (2024).

[36] Shi, Z. J. et al. Continuous angle-tunable birefringence with freeform metasurfaces for arbitrary polarization conversion. *Sci. Adv.* **6**, eaba3367 (2020). 32537506 10.1126/sciadv.aba3367 PMC7269657

[37] Wang, S. et al. Metasurface-based solid Poincaré sphere polarizer. *Phys. Rev. Lett.* **130**, 123801 (2023). 37027878 10.1103/PhysRevLett.130.123801

[38] Chen, T. Q. et al. Freeform metasurface-assisted waveguide coupler for guided wave polarization manipulation and spin-orbit angular momentum conversion. *ACS Photonics* **11**, 1051–1059 (2024).

[39] Chen, Y. Q. et al. Topology optimization-based inverse design of plasmonic nanodimer with maximum near-field enhancement. *Adv. Funct. Mater.* **30**, 2000642 (2020).

[40] Zhao, Y. G. et al. Fast topology optimization for near-field focusing all-dielectric metasurfaces using the discrete dipole approximation. *ACS Nano* **16**, 18951–18958 (2022). 36314904 10.1021/acsnano.2c07848

[41] Nam, S. H. et al. Photolithographic realization of target nanostructures in 3D space by inverse design of phase modulation. *Sci. Adv.* **8**, eabm6310 (2022). 35613258 10.1126/sciadv.abm6310 PMC9132447

[42] Xu, M. F. et al. Emerging long-range order from a freeform disordered metasurface. *Adv. Mater.* **34**, 2108709 (2022). 10.1002/adma.202108709 34997941

[43] Cordaro, A. et al. Solving integral equations in free space with inverse-designed ultrathin optical metagratings. *Nat. Nanotechnol.* **18**, 365–372 (2023). 36635333 10.1038/s41565-022-01297-9

[44] Mann, S. A., Goh, H. & Alù, A. Inverse design of nonlinear polaritonic metasurfaces for second harmonic generation. *ACS Photonics* **10**, 993–1000 (2023).

[45] Willets, K. A. et al. Super-resolution imaging and plasmonics. *Chem. Rev.* **117**, 7538–7582 (2017). 28084729 10.1021/acs.chemrev.6b00547

[46] Hong, F. & Blaikie, R. Plasmonic lithography: Recent progress. *Adv. Opt. Mater.* **7**, 1801653 (2019).

[47] Luo, X. G. & Ishihara, T. Surface plasmon resonant interference nanolithography technique. *Appl. Phys. Lett.* **84**, 4780–4782 (2004).

[48] Fang, N. et al. Sub-diffraction-limited optical imaging with a silver superlens. *Science* **308**, 534–537 (2005). 15845849 10.1126/science.1108759

[49] Zhang, X. & Liu, Z. W. Superlenses to overcome the diffraction limit. *Nat. Mater.* **7**, 435–441 (2008). 18497850 10.1038/nmat2141

[50] Luo, X. G. Principles of electromagnetic waves in metasurfaces. *Sci. China Phys. Mech. Astron.* **58**, 594201 (2015).

[51] Xu, T. et al. Localizing surface plasmons with a metal-cladding superlens for projecting deep-subwavelength patterns. *Appl. Phys. B* **97**, 175–179 (2009).

[52] Yang, X. F. et al. Breaking the feature sizes down to sub-22 nm by plasmonic interference lithography using dielectric-metal multilayer. *Opt. Express* **17**, 21560–21565 (2009). 19997397 10.1364/OE.17.021560

[53] Gao, P. et al. Enhancing aspect profile of half-pitch 32 nm and 22 nm lithography with plasmonic cavity lens. *Appl. Phys. Lett.* **106**, 093110 (2015).

[54] Liu, H. et al. High contrast superlens lithography engineered by loss reduction. *Adv. Funct. Mater.* **22**, 3777–3783 (2012).

[55] Chen, X. et al. Large-area high aspect ratio plasmonic interference lithography utilizing a single high-k mode. *ACS Nano* **10**, 4039–4045 (2016). 27075440 10.1021/acsnano.5b06137

[56] Luo, Y. F. et al. Proximity correction and resolution enhancement of plasmonic lens lithography far beyond the near field diffraction limit. *RSC Adv.* **7**, 12366–12373 (2017).

[57] Guan, F. X. et al. Overcoming losses in superlenses with synthetic waves of complex frequency. *Science* **381**, 766–771 (2023). 37590345 10.1126/science.adi1267

[58] Pendry, J. B. Negative refraction makes a perfect lens. *Phys. Rev. Lett.* **85**, 3966–3969 (2000). 11041972 10.1103/PhysRevLett.85.3966

[59] Pendry, J. B. & Ramakrishna, S. A. Refining the perfect lens. *Phys. B: Condens. Matter* **338**, 329–332 (2003).

[60] Luo, J. et al. Fabrication of anisotropically arrayed nano-slots metasurfaces using reflective plasmonic lithography. *Nanoscale* **7**, 18805–18812 (2015). 26507847 10.1039/c5nr05153c

[61] Liu, L. Q. et al. Batch fabrication of metasurface holograms enabled by plasmonic cavity lithography. *Adv. Opt. Mater.* **5**, 1700429 (2017).

[62] Wong, A. K.-K. *Optical imaging in projection microlithography* (SPIE Press, 2005).

[63] Yeh, P. & Hendry, M. Optical waves in layered media. *Phys. Today* **43**, 77–78 (1990).

[64] Zhan, T. R. et al. Transfer matrix method for optics in graphene layers. *J. Phys. Condens. Matter* **25**, 215301 (2013). 23628895 10.1088/0953-8984/25/21/215301

[65] Ma, X. & Arce, G. R. *Computational Lithography*. (Hoboken: John Wiley & Sons, 2010).

[66] Dainese, P. et al. Shape optimization for high efficiency metasurfaces: theory and implementation. *Light Sci. Appl.* **13**, 300 (2024). 39468011 10.1038/s41377-024-01629-5 PMC11519467

[67] Shen, Y. J., Wong, N. & Lam, E. Y. Level-set-based inverse lithography for photomask synthesis. *Opt. Express* **17**, 23690–23701 (2009). 20052080 10.1364/OE.17.023690

[68] Ma, X., Li, Y. Q. & Dong, L. S. Mask optimization approaches in optical lithography based on a vector imaging model. *J. Opt. Soc. Am. A* **29**, 1300–1312 (2012). 10.1364/JOSAA.29.001300 22751396

[69] Ma, X. et al. Nonlinear compressive inverse lithography aided by low-rank regularization. *Opt. Express* **27**, 29992–30008 (2019). 31684254 10.1364/OE.27.029992

[70] Chung, H. et al. Inverse design of high-NA metalens for maskless lithography. *Nanophotonics* **12**, 2371–2381 (2023). 39633747 10.1515/nanoph-2022-0761 PMC11501502

[71] Yang, Y. M. et al. All-dielectric metasurface analogue of electromagnetically induced transparency. *Nat. Commun.* **5**, 5753 (2014). 25511508 10.1038/ncomms6753

[72] Ma, W. et al. Deep learning for the design of photonic structures. *Nat. Photonics* **15**, 77–90 (2021).

[73] Ma, T. et al. Benchmarking deep learning-based models on nanophotonic inverse design problems. *Opto-Electron. Sci.* **1**, 210012 (2022).

[74] Ma, T. G., Wang, H. Z. & Guo, L. J. OptoGPT: a foundation model for inverse design in optical multilayer thin film structures. *Opto-Electron. Adv.* **7**, 240062 (2024).

[75] Liu, M. Z. et al. Multifunctional metasurfaces enabled by simultaneous and independent control of phase and amplitude for orthogonal polarization states. *Light Sci. Appl.* **10**, 107 (2021). 34035215 10.1038/s41377-021-00552-3 PMC8149653

[76] Song, M. W. et al. Versatile full-colour nanopainting enabled by a pixelated plasmonic metasurface. *Nat. Nanotechnol.* **18**, 71–78 (2023). 36471110 10.1038/s41565-022-01256-4

[77] Clark, L., Roberts, A. & Wesemann, L. Neural reparameterization for nonlocal metasurface topology optimization. *APL Photonics* **10**, 100803 (2025).

[78] Hammond, A. M. et al. Unifying and accelerating level-set and density-based topology optimization by subpixel-smoothed projection. *Opt. Express* **33**, 33620–33642 (2025). 40984522 10.1364/OE.563512

