# Supplementary material for: Full-space inverse-designed meta-optics for complex vector field shaping of intracavity landscapes
Source: Light Sci Appl. 2026 Apr 3;15:187. doi: 10.1038/s41377-026-02258-w (PMC13049160; doi:10.1038/s41377-026-02258-w)
Supplement: Supplementary file 1 — Supporting Information for Full-space inverse-designed meta-optics for complex vector field shaping of intracavity landscapes. [file 41377_2026_2258_MOESM1_ESM.pdf]

Supplemental Information for

Full-space inverse-designed meta-optics for complex vector field shaping of intracavity landscapes

Mingfeng Xu[1,2,3,4†], Di Sang[1,5†], Mingbo Pu[1,2,3,4✉], Ping Gao[1,2,4], Bowen Zhao[1,2], Yuhan Zheng[1,2,3,4], Qingji Zeng[1,2,3], Lianwei Chen[1,2,3], Fei Zhang[1,2,3], Yinghui Guo[1,2,3,4], Xiong Li[1,2,4], Xiaoliang Ma[1,2,4], Yunqi Fu[5], and Xiangang Luo[1,2,4✉]

[1]National Key Laboratory of Optical Field Manipulation Science and Technology, Chinese Academy of Sciences, Chengdu 610209, China.

[2]State Key Laboratory of Optical Technologies on Nano-Fabrication and Micro-Engineering, Institute of Optics and Electronics, Chinese Academy of Sciences, Chengdu 610209, China.

[3]Research Center on Vector Optical Fields, Institute of Optics and Electronics, Chinese Academy of Sciences, Chengdu 610209, China.

[4]College of Materials Science and Opto-Electronic Technology, University of Chinese Academy of Sciences, Beijing 100049, China.

[5]College of Electronic Science and Technology, National University of Defense Technology, Changsha 410073, China.

[†]These authors contributed equally to this work.

[✉]email: pmb@ioe.ac.cn, lxg@ioe.ac.cn.

Supplementary Note 1. The adjoint simulation method.

The function of merit depends on the field values at regions within the target domain (Ω), which can be written as:

$$F(\mathbf{E}(\mathbf{x_0})) = \int_{x_0 \in \Omega} f(\mathbf{E}(\mathbf{x_0}))dx_0 \tag{S1}$$

where $f(\mathbf{E}(\mathbf{x_0}))$ represents a local error in the target domain at the point $x_0 \in \Omega$. The functional derivative of F is usually used to describe variation in F with respect to slight changes in design structure. The variation in F depends on the independent variations $\partial\mathbf{E}$ and $\partial\mathbf{E}^*$ and consequently can be written as

$$\delta F = \int_{x_0 \in \Omega} [\frac{\partial f}{\partial \mathbf{E}} \cdot \partial \mathbf{E} + \frac{\partial f}{\partial \mathbf{E}^*} \cdot \partial \mathbf{E}^*] d\mathbf{x_0} \tag{S2}$$

The variation in the electric $\partial\mathbf{E}$ at x_0 is derived from the interaction of the induced polarization density $\mathbf{P^{ind}}(\mathbf{x}')$:

$$\delta \mathbf{E} = \int_{\mathbf{x}' \in \chi} \overline{\overline{G^{EP}}}(\mathbf{x_0}, \mathbf{x}')\mathbf{P^{ind}}(\mathbf{x}')d\mathbf{x}' \tag{S3}$$

where $\overline{\overline{G^{EP}}}(\mathbf{x_0}, \mathbf{x}')$ is the tensor containing the electric field at x_0 from a unit electric dipole at $\mathbf{x}'$ and χ is the design domain.

When the dielectric constant changes slightly such that $\varepsilon(\mathbf{x}') \to \varepsilon(\mathbf{x}') + \delta\varepsilon$, it is reasonable to assume that there is no significant change in the electric field at this point. By Born approximation, its induced polarization density is

$$\mathbf{P^{ind}}(\mathbf{x}') \simeq \delta\varepsilon \mathbf{E_{old}}(\mathbf{x}') \tag{S4}$$

At this time, the variation of F can be expressed as

$$\delta F = \int_{\mathbf{x}' \in \chi} 2\Re[\int_{x_0 \in \Omega} \overline{\overline{G^{EP}}}(\mathbf{x}', \mathbf{x_0})\frac{\partial f}{\partial \mathbf{E}}(\mathbf{x_0})d\mathbf{x_0} \cdot \delta\varepsilon\delta V \mathbf{E_{old}}(\mathbf{x}')]d\mathbf{x}' \tag{S5}$$

We can then define a new term:

$$\mathbf{E_{adj}}(\mathbf{x}') = \int_{x_0 \in \Omega} \overline{\overline{G^{EP}}}(\mathbf{x}', \mathbf{x_0})\frac{\partial f}{\partial \mathbf{E}}(\mathbf{x_0})d\mathbf{x_0} \tag{S6}$$

where $\partial f/\partial\mathbf{E}(\mathbf{x_0})$ can be regarded as the excitation source of $\mathbf{E_{adj}}(\mathbf{x}')$, also known as the adjoint source. In this paper, we choose the dielectric constant within the

design domain as the design variables. The optimization gradient requires to be expressed as the variation of FoM in terms of the variation of dielectric constant. We define our function of merit to be:

$$\frac{\partial F}{\partial \varepsilon} = \int_{\mathbf{x}' \in \chi} 2\Re[\mathbf{E_{adj}}(\mathbf{x}') \cdot \mathbf{E_{old}}(\mathbf{x}')] d\mathbf{x}' \tag{S7}$$

Supplementary Note 2. Different configurations of SPP near-field imaging

Different configurations of SPP near-field imaging system are presented in Fig. S1. In comparison with the conventional near-field imaging system (Fig. S1a), the perfect superlens configuration more high-frequency components in the photoresist film (Fig. S1b). For the plasmonic reflective (Fig. S1c) and cavity superlens (Fig. S1d) configurations, both the corresponding electric fields in the photoresist film are superior to the perfect superlens in terms of longitudinal fidelity of the high-frequency components. Particularly, the cavity superlens configuration has the best performance for near-field imaging, while the introducing of additional metal films increases the complexity and process difficulty.

Here, the plasmonic reflective superlens configuration is adopted as the SPP near-field imaging system to demonstrate the fidelity enhancement performance of the proposed optimization method. As shown in shown in Fig. S1c, it consists of a metasurface mask, an air spacer, a photoresist film, and a metal reflective film. Specifically, the metal reflective film is served as a hard mask for subsequent pattern transfer to a substrate or a thicker polymer resist layer, while its thickness has less impact on the system (see Fig. S2). In principle, the thickness of the photoresist-air spacer needs to be traded off according to the process conditions (e.g., the thickness of target material and deposition method) to allow more high-frequency evanescent waves to enter the photoresist while suppressing the E_z component (see Fig. S4a). For example, although a thick photoresist facilitates easier etch or lift-off, it causes

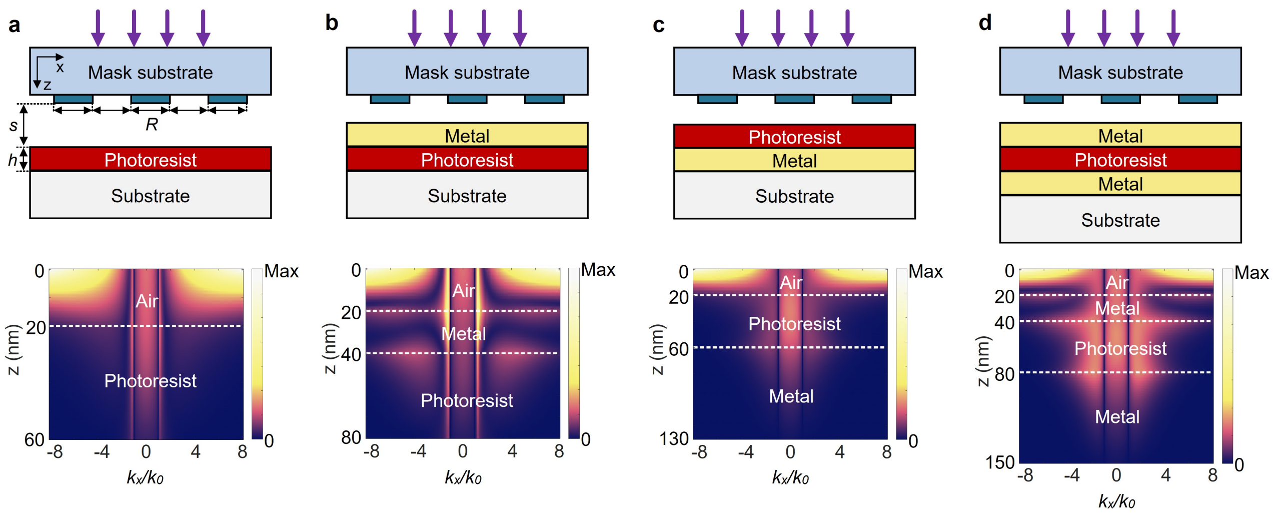

Figure S1 | Cross–section of typical near-field imaging systems configuration and longitudinal spectral transmissions as a function of the normalized spatial frequency vector k_x/k_0. (**a**) Traditional near-field configuration, (**b**) perfect superlens configuration, (**c**) plasmonic reflective superlens configuration, and (**d**) plasmonic cavity superlens configuration. Electric field distributions are shown for TM polarization.

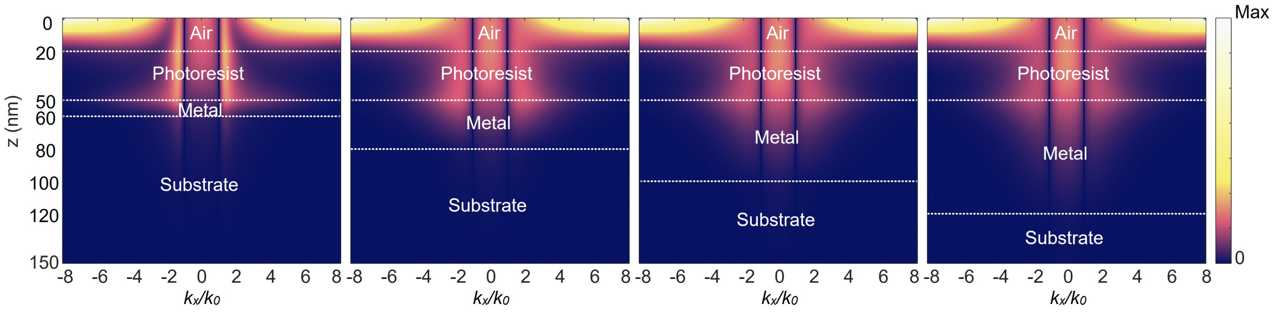

Figure S2 | Effect of metal reflective layer thickness on the electric field distribution in the reflective plasmonic near-field imaging system. Theoretically, the metal reflective layer thickness needs to be more higher than the skinning depth. The field distribution is simulated under TM illumination.

diffraction and poor longitudinal fidelity inside the photoresist film. In our work, the thicknesses of the air spacer, photoresist and metal film are set to 20 nm, 30 nm and 70 nm, respectively, with optimized modifications.

Supplementary Note 3. Transfer matrix method for SPP near-field imaging

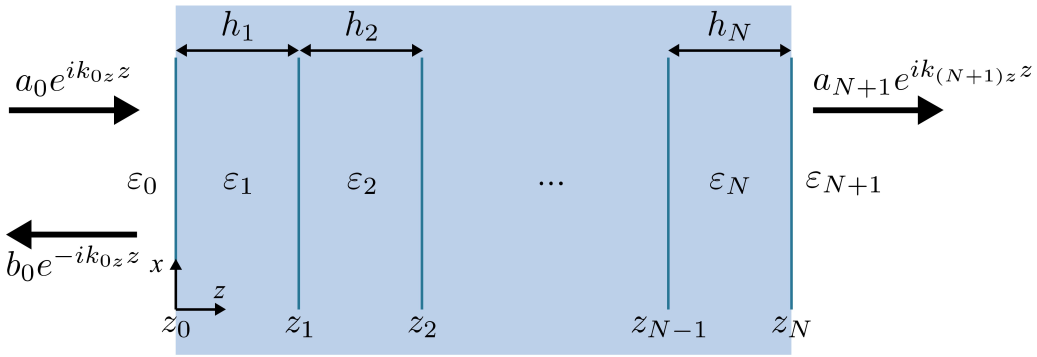

Figure S3 | Schematic of a multilayer film system. The electromagnetic field can be represented as forward and reverse transmission waves in each layer. a_i and b_i represent the amplitude of the right-traveling and left-traveling wave, respectively. h_i indicate the thickness of each layer and ε_i is the dielectric constants. The structure is coupled to homogeneous mediums characterized by ε_0 (initial medium) and ε_{N+1} (final medium).

In the Hopkins imaging model, the image is formed by the combined contributions of the metasurface mask pattern and the optical system in spectral and spatial form:

$$\mathbf{E}_{\text{print}}(x, y) = \mathcal{F}^{-1}\{\mathbf{G}(k_x, k_y) \otimes M(k_x, k_y) \cdot \text{OTF}(k_x, k_y)\} \tag{S8}$$

where $\mathbf{E}_{\text{print}}(x, y)$ represents the electric field in the image plane, $\mathbf{G}(k_x, k_y)$ and $M(k_x, k_y)$ indicate the illumination function and the metasurface mask spectrum for different Fourier components, respectively. $\otimes$ denotes the convolution operation, representing the filtering process of the mask spectrum by the optical system. $\mathcal{F}^{-1}\{\cdot\}$ denotes the inverse Fourier transform and $\text{OTF}(k_x, k_y)$ is the optical transfer function of the imaging system, which can be obtained by the transfer matrix method (TMM).

Assuming that the coupling interaction between the plasmonic superlens and the metasurface mask is neglected, the three-component field propagates in the three-dimensional space of an isotropic homogeneous medium. Therefore, the plasmonic near-field imaging process can be simplified to the problem of reflection and transmission of electromagnetic radiation through a multilayer film. Without loss of generality, we consider a one-dimensional structure consisting of homogeneous medium layers characterized by different dielectric constants ε_i $(i = 0, 1, ...N, N+1)$. Figure S3 schematically shows this structure, where h_i is the thickness of each layer, and ε_0 and ε_{N+1} are the dielectric constants of the incident medium and the substrate, respectively. The TMM allows for the correlation of the electric and magnetic fields at different locations in the layered dielectric medium through different matrices, including the transmission matrix connecting the fields through the interface

and the propagation matrix connecting the fields propagating some distance within the homogeneous medium.

For the multilayer structure considered here, transverse magnetic (TM) and transverse electric (TE) polarization can be decoupled and dealt with separately. For TM polarization, the electric field is polarized along the y-direction and can be written in the form of a superposition of propagating waves in the $+z$ and $-z$ direction:

$$H_{iy}(x,z) = (a_i e^{jk_{iz}z} + b_i e^{-jk_{iz}z})e^{jk_{ix}x}, z_{i-1} < z < z_i \tag{S9}$$

where a_i and b_i represent the field coefficients, $k_{ix(z)}$ is the $x(z)$ component of the wave vector k_i ($k_i = \sqrt{\varepsilon_i}\omega/c$), ω is the angular frequency, and c is the speed of light in vacuum.

With $\nabla \times \mathbf{H} = j\omega\varepsilon\mathbf{E}$, the electric fields can be written as:

$$\begin{cases} E_{ix} = \frac{k_{iz}}{\omega\varepsilon_i}(-a_i e^{jk_{iz}z} + b_i e^{-jk_{iz}z})e^{jk_{ix}x} \\ E_{iz} = \frac{k_{ix}}{\omega\varepsilon_i}(a_i e^{jk_{iz}z} + b_i e^{-jk_{iz}z})e^{jk_{ix}x} \end{cases} \tag{S10}$$

From the phase matching condition of Snells law, we will immediately have the same lateral propagation constant $k_{ix} = k_x$. The electric and magnetic fields at the interface satisfy the following boundary conditions:

$$\begin{cases} \mathbf{n} \times (\mathbf{H_1} - \mathbf{H_0})|_{z=z_0} = 0 \\ \mathbf{n} \times (\mathbf{E_1} - \mathbf{E_0})|_{z=z_0} = 0 \end{cases} \tag{S11}$$

where $\mathbf{n}$ is the unit surface normal.

Applying the above boundary conditions at $z = 0$, the coefficients a_0 and b_0 can be related to a_1 and b_1 by a 2×2 transmission matrix $D_{0\to1}$:

$$\begin{bmatrix} a_0 \\ b_0 \end{bmatrix} = D_{0\to1} \begin{bmatrix} a_1 \\ b_1 \end{bmatrix} \tag{S12}$$

where $D_{0\to1} = \frac{1}{2}\begin{bmatrix} 1+\eta & 1-\eta \\ 1-\eta & 1+\eta \end{bmatrix}$ with the parameters $\eta = \frac{\varepsilon_0 k_{1z}}{\varepsilon_1 k_{0z}}$. Similarly, for TE polarization of the electric field along y-direction, the transmission matrix has the identical form while with the parameter $\eta = \frac{k_{1z}}{k_{0z}}$. As for the field correlation propagation

matrix in a homogeneous medium, it can be expressed as:

$$P(h) = \begin{bmatrix} e^{-ik_zh} & 0 \\ 0 & e^{ik_zh} \end{bmatrix} \tag{S13}$$

Therefore, the left-side field and right-side field coefficients can be related by the 2×2 transfer matrix M $[M = D_{0\to1}P(h_1)D_{1\to2}...P(h_N)D_{N\to N+1}]$ as:

$$\begin{bmatrix} a_0 \\ b_0 \end{bmatrix} = M \begin{bmatrix} a_{N+1} \\ b_{N+1} \end{bmatrix} \tag{S14}$$

We note that a_0 and b_{N+1} are the incident waves, while b_0 and a_{N+1} are the outgoing waves as unknown quantities. From the transfer matrix, it is easy to obtain the scattering matrix of the associated incident and outgoing plane waves:

$$\begin{bmatrix} b_0 \\ a_{N+1} \end{bmatrix} = S \begin{bmatrix} a_0 \\ b_{N+1} \end{bmatrix} \tag{S15}$$

where the scattering matrix $S = \begin{bmatrix} M_{21}/M_{11} & (M_{11}M_{22}-M_{12}M_{21})/M_{11} \\ 1/M_{11} & -M_{12}/M_{11} \end{bmatrix}$, and M_{ij} is the element of M.

Supplementary Note 4. Full-vectorial analysis of plasmonic cavity

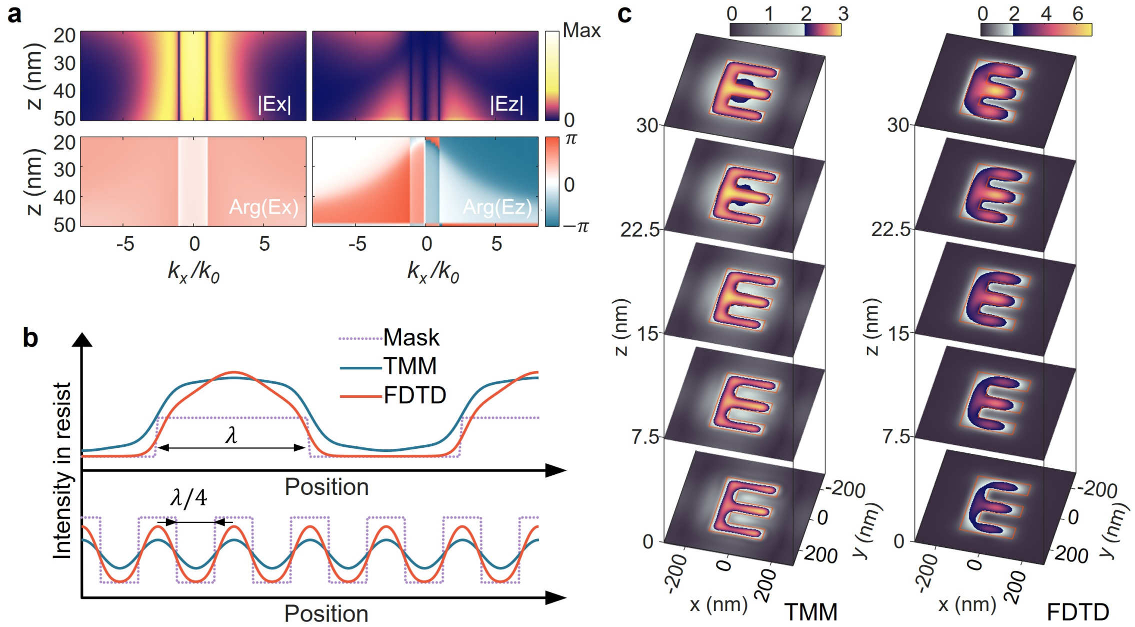

Figure S4 | (**a**) Transverse $|E_x|$ and longitudinal $|E_z|$ electric field amplitude and phase at the photoresist film. k_x/k_0 is the normalized wavevector along the x direction. (**b**) Intensity distribution in the middle location of photoresist film, with metasurface mask density $R = \lambda$ and $R = \lambda/4$. (**c**) Cross-section view of normalized optical intensity distribution in the photoresist region calculated by transfer matrix method (TMM) and finite-difference time-domain (FDTD) method, respectively. The intensity above and below the threshold are represented in different colormaps to visualize the binarized pattern profile.

As shown in Fig. S4a, the E_x component has a homogeneous longitudinal distribution, which is helpful to realize the super-resolution imaging. In contrast, the E_z component exhibits pronounced variations in both amplitude and phase along the z-axis. This longitudinal non-uniformity of E_z can significantly degrade the imaging fidelity in the vertical dimension, leading to inevitable pattern distortions in the photoresist. Specifically, the linear physical model of transfer matrix method (TMM) ignores the coupling interaction between the plasmonic reflective superlens and the metasurface mask. As a result, considering such complex subwavelength electromagnetic mechanism, full modeling method should be adopted to accurately calculate the actual imaging patterns. For example, Fig. S4b presents the large discrepancy of grating imaging results between TMM and the finite-difference time-domain method (FDTD). It is worth noting that near-field lithography can only achieve a resolution of 0.46λ in this configuration, while plasmonic near-field imaging system performs well in the case of $\lambda/4$ gratings, despite the poor rectangularity of the image due to the loss of very high-frequency information. Figure S4c shows the normalized intensity distributions $|\mathrm{E}|^2$ calculated by TMM and FDTD method for a typical "E"

image. It is revealed that, in comparison with FDTD method, the TMM model introduces significant distortions both in the corner pattern and the longitudinal fidelity. Therefore, it is difficult to accurately characterize the full-vectorial imaging pattern in such super-resolution optical near-field imaging system with conventional linear imaging model.

Supplementary Note 5. Full-space adjoint method for the fidelity optimization of SPP near-field imaging

Here, the figure of merit (FoM) for the topology optimization of the optical imaging fidelity depends on the field values at regions within the target domain (Ω), which can be written as:

$$\text{FoM} = \int_{\mathbf{x}_0 \in \Omega} f(\mathbf{x}_0) d\mathbf{x}_0 = \int_{\mathbf{x}_0 \in \Omega} \parallel P_d(\mathbf{x}_0) - P_a(\mathbf{E}(\mathbf{x}_0)) \parallel_2^2 d\mathbf{x}_0 \tag{S16}$$

where $f(\mathbf{x}_0)$ represents a local error in the target domain at point $\mathbf{x_0} \in \Omega$. $P_d(\mathbf{x}_0)$ represents the object pattern and $P_a(\mathbf{E}(\mathbf{x}_0))$ represents the actual pattern, which are expressed as:

$$P_d(\mathbf{x}_0) = \begin{cases} 0, \mathbf{x}_0 \notin \text{desired pattern region} \\ 1, \mathbf{x}_0 \in \text{desired pattern region} \end{cases} \tag{S17}$$

$$P_a(\mathbf{E}(\mathbf{x}_0)) = \begin{cases} 0, |\mathbf{E}(\mathbf{x}_0)|^2 < I_{th} \\ 1, |\mathbf{E}(\mathbf{x}_0)|^2 \geqslant I_{th} \end{cases} \tag{S18}$$

where I_{th} represents the threshold of photoresist.

Then, due to independent dual-polarized optical excitation of the TM and TE, the variation in FoM could be expressed as the independent variations of $\delta\mathbf{E}_{\text{TM}}$, $\delta\mathbf{E}^*_{\text{TM}}$, $\delta\mathbf{E}_{\text{TE}}$ and $\delta\mathbf{E}^*_{\text{TE}}$ as:

$$\begin{aligned} \delta FoM &= \textstyle\int_\Omega [\frac{\delta f}{\delta \mathbf{E}_{\text{TM}}} \cdot \delta\mathbf{E}_{\text{TM}} + \frac{\delta f}{\delta \mathbf{E}^*_{\text{TM}}} \cdot \delta\mathbf{E}^*_{\text{TM}} + \frac{\delta f}{\delta \mathbf{E}_{\text{TE}}} \cdot \delta\mathbf{E}_{\text{TE}} + \frac{\delta f}{\delta \mathbf{E}^*_{\text{TE}}} \cdot \delta\mathbf{E}^*_{\text{TE}}] d\mathbf{x}_0 \\ &= 2\Re\left[\textstyle\int_\Omega (\frac{\delta f}{\delta \mathbf{E_{TM}}} \cdot \delta\mathbf{E_{TM}} + \frac{\delta f}{\delta \mathbf{E_{TE}}} \cdot \delta\mathbf{E_{TE}}) d\mathbf{x}_0\right] \end{aligned} \tag{S19}$$

To calculate the gradient information, the FoM of imaging fidelity could be rewritten as:

$$f(\mathbf{E}_{\text{TM}}, \mathbf{E}_{\text{TE}}) = |P_d - Pa|^2 \cong (P_d - {P_a}')^2 \tag{S20}$$

where

$$P_a \approx \frac{1}{1 + \exp[-A(|\mathbf{E_{TM}}|^2 + |\mathbf{E_{TE}}|^2 - I_{th})]} \equiv {P_a}' \tag{S21}$$

Then, the partial derivative of local FOM with respect to the two independent electric fields is:

$$\begin{cases} \dfrac{\delta f(\mathbf{E}_{\text{TM}}, \mathbf{E}_{\text{TE}})}{\delta \mathbf{E}_{\text{TM}}} = 2(P_d - P_a)(-\dfrac{\delta P_a}{\delta \mathbf{E}_{\text{TM}}}) \\ \dfrac{\delta f(\mathbf{E}_{\text{TM}}, \mathbf{E}_{\text{TE}})}{\delta \mathbf{E}_{\text{TE}}} = 2(P_d - P_a)(-\dfrac{\delta P_a}{\delta \mathbf{E}_{\text{TE}}}) \end{cases} \tag{S22}$$

where

$$\begin{aligned}\frac{\delta P_a}{\delta \mathbf{E}_{\text{TM}}} &\approx \frac{-\exp[-A(|\mathbf{E}_{\text{TM}}|^2+|\mathbf{E}_{\text{TE}}|^2-I_{\text{th}})]}{(1+\exp[-A(|\mathbf{E}_{\text{TM}}|^2+|\mathbf{E}_{\text{TE}}|^2-I_{\text{th}})])^2}(-A\frac{\delta|\mathbf{E}_{\text{TM}}|^2}{\delta \mathbf{E}_{\text{TM}}}) \\ &= A\exp[-A(|\mathbf{E}_{\text{TM}})|^2+|\mathbf{E}_{\text{TE}})|^2-I_{\text{th}})]{P_a}^2\frac{\delta|\mathbf{E}_{\text{TM}}|^2}{\delta \mathbf{E}_{\text{TM}}}\end{aligned} \tag{S23}$$

Because $\delta|\mathbf{E}_{\text{TM}}|^2/\delta\mathbf{E}_{\text{TM}} = \mathbf{E}^*_{\text{TM}}$, we can get:

$$\frac{\delta P_a}{\delta \mathbf{E}_{\text{TM}}} \approx A\exp[-A(|\mathbf{E}_{\text{TM}})|^2+|\mathbf{E}_{\text{TE}})|^2-I_{\text{th}})]{P_a}^2\mathbf{E}^*_{\text{TM}} \tag{S24}$$

Similarly,

$$\frac{\delta P_a}{\delta \mathbf{E}_{\text{TE}}} \approx A\exp[-A(|\mathbf{E}_{\text{TM}})|^2+|\mathbf{E}_{\text{TE}})|^2-I_{\text{th}})]{P_a}^2\mathbf{E}^*_{\text{TE}} \tag{S25}$$

Therefore, the partial derivatives of local FOM, i.e., the adjoint source, are expressed as:

$$\begin{cases}\dfrac{f(\mathbf{E}_{\text{TM}},\mathbf{E}_{\text{TE}})}{\delta \mathbf{E}_{\text{TM}}} = 2A(P_a-P_d){P'_a}^2\exp[-A(|\mathbf{E}_{\text{TM}}|^2+|\mathbf{E}_{\text{TE}}|^2-I_{\text{th}})]\mathbf{E}^*_{\text{TM}} \\ \dfrac{f(\mathbf{E}_{\text{TM}},\mathbf{E}_{\text{TE}})}{\delta \mathbf{E}_{\text{TE}}} = 2A(P_a-P_d){P'_a}^2\exp[-A(|\mathbf{E}_{\text{TM}}|^2+|\mathbf{E}_{\text{TE}}|^2-I_{\text{th}})]\mathbf{E}^*_{\text{TE}}\end{cases} \tag{S26}$$

Supplementary Note 6. Analysis of image quality under angular misalignments in the cavity assembly

Figure S5 illustrates the mask imaging qualities before and after optimization under different oblique incidence angles ($\theta = -10^\circ, -5^\circ, 0^\circ, 5^\circ, 10^\circ; \phi\ = 0^\circ$). The contour lines indicate the binary threshold of the exposed and developed pattern, while the dashed lines outline the standard "E" shape. The results show that, as the incident angle increases from negative to positive values, the imaging performance of both the initial and optimized masks degrades to some extent. However, the optimized mask consistently maintains a contour close to the standard "E" shape across all angles, demonstrating significantly better preservation of edge sharpness and shape integrity, especially at the 90° corners, compared to the initial mask. Specifically, the initial mask exhibits noticeable lateral compression and unstable corner shapes as the angle varies, whereas the optimized mask shows superior overall shape consistency. Nonetheless, under certain angles ($-10^\circ$and 10°), distortions such as bending or even breaks in the middle horizontal bar still occur, indicating that while the current optimization has improved angular robustness, it has not fully overcome local imaging distortions induced by oblique incidence. Furthermore, this issue can be addressed by employing a multiobjective optimization approach within the topology optimization framework. By systematically minimizing the worstcase imaging performance across a range of incident angles, process variations caused by angular fluctuations can be reduced, thereby achieving more robust crossangle imaging performance in practical fabrication processes.

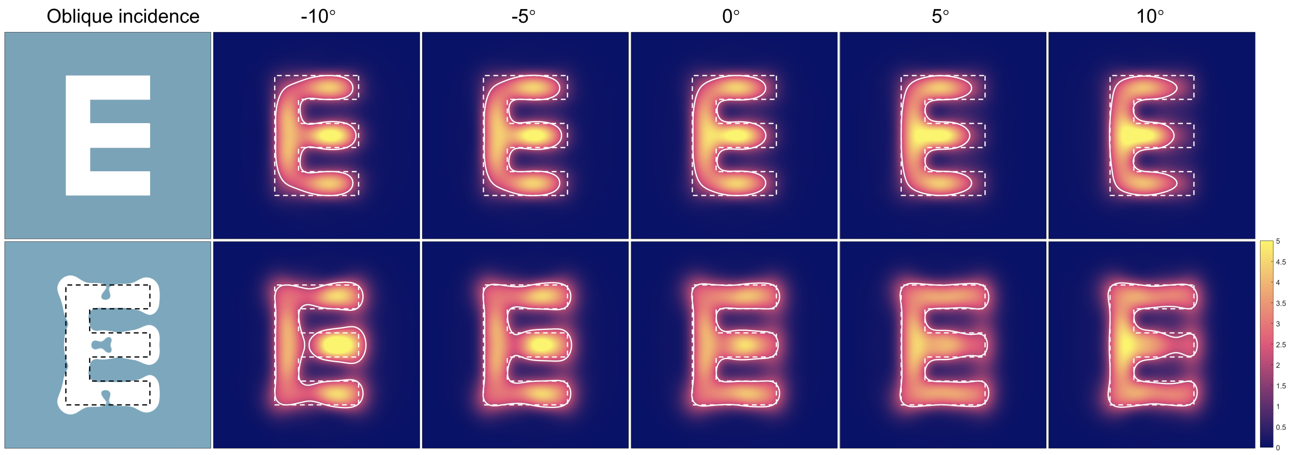

Figure S5 | Robustness analysis against oblique incidence. Simulated imaging results for the initial (first row) and optimized (second row) mask under different in-plane oblique incidence angles ($\theta = -10^\circ, -5^\circ, 0^\circ, 5^\circ, 10^\circ; \phi\ = 0^\circ$).

Supplementary Note 7. Analysis of image quality under fabrication errors

Figure S6 shows the simulated imaging intensity distribution for the optimized E-shaped mask when its transparent regions undergo different dimensional deviations (expansion or shrinkage by 5 nm and 10 nm). Analysis indicates that mask shrinkage reduces optical throughput and narrows the printed linewidth, while expansion has the opposite effect. Despite these systematic changes in intensity and linewidth, high-fidelity pattern transfer can still be achieved within a reasonable tolerance range by co-optimizing exposure dose and development threshold. However, larger fabrication errors will lead to irreversible geometric distortions consistent with the mask deformation trend. To systematically improve tolerance to manufacturing variations, a multi-objective topology optimization strategy could be incorporated in future work, aiming to minimize the worst-case imaging performance across a set of predefined error scenarios.

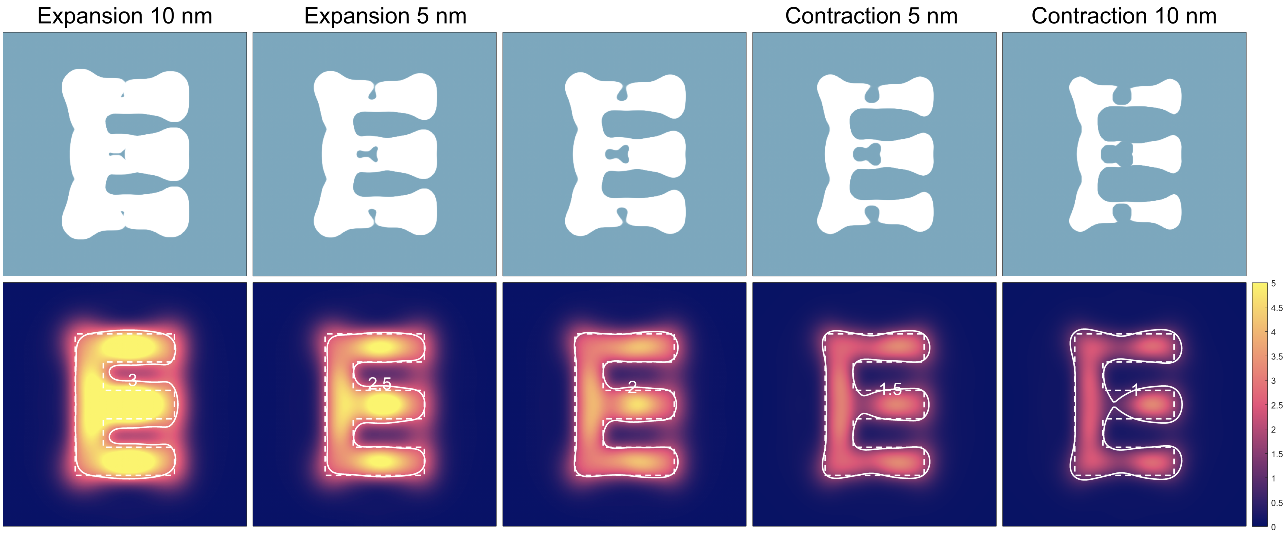

Figure S6 | Robustness analysis against fabrication errors. Simulated imaging intensity distribution for the optimized E-shaped mask under different dimensional deviations (from left to right: expansion by +10 nm, +5 nm, nominal size, shrinkage by -5 nm, -10 nm). The contour lines (from left to right) correspond to normalized intensity levels of 3, 2.5, 2, 1.5, and 1, illustrating the exposure-dose response under different mask deviations.

Supplementary Note 8. Comparative analysis of full-space vs. semi-space adjoint methods

To quantitatively substantiate the necessity and advantage of full-space adjoint formulation, we performed a direct and comparative analysis for the "E" pattern within our plasmonic cavity system (Fig. 3A). Two separate optimization simulations were conducted: one using our proposed full-space adjoint method (accounting for both $k_z > 0$ and $k_z < 0$ modes) and another using a conventional semi-space adjoint method (considering only outgoing waves with $k_z > 0$ in the adjoint simulation). The evolution processes of optimized Cr metasurface mask morphology and near-field imaging patterns for semi-space adjoint (using only backward adjoint source) and full-space adjoint (using bidirectional adjoint sources) method are shown in the Fig. S7. Moreover, the different convergence behaviors are shown in the Fig. S8. Both methods exhibit a similar rapid decrease in the Area Error Ratio (AER) during the initial 80 iterations. However, a decisive divergence occurs thereafter. The semi-space method shows markedly poor robustness during the later stages of binarization and fine-tuning, causing its AER to plateau at a significantly higher value (1.7%) compared to the full-space method (1%). This indicates that the gradient information provided by the semi-space adjoint becomes incomplete or misleading when strong multiple reflections within the enclosed cavity must be managed simultaneously with drastic topological changes (e.g., binarization).

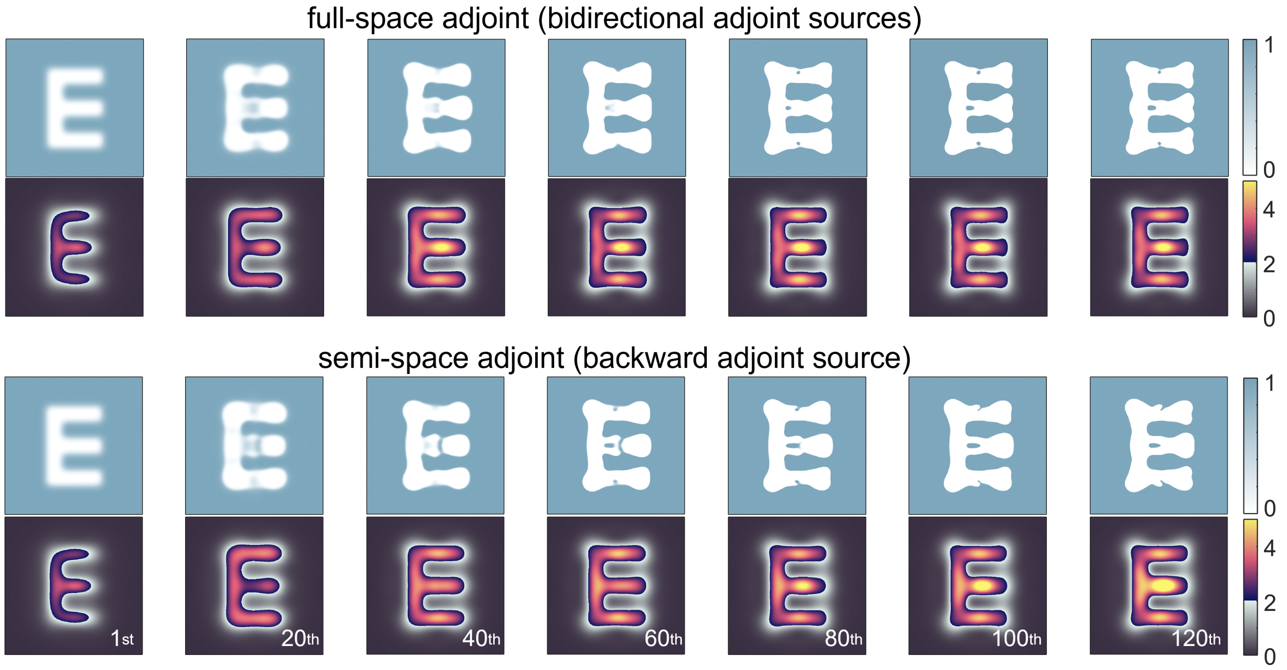

Figure S7 | Evolution process of optimized Cr metasurface mask morphology and near-field imaging patterns during the optimization iterations.

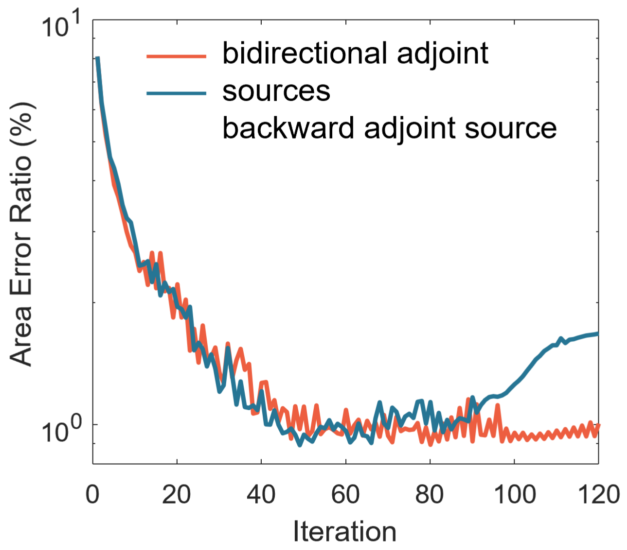

Figure S8 | Evolution of the Area Error Ratio (AER) during topology optimization for the "E" pattern using the full-space adjoint method (blue) and the semi-space adjoint method (red line).

For the specific multilayer cavity studied here, where the target plane lies within the resonant gap, the primary limitation of the semi-space approach manifests not in the early convergence rate but in its ultimate convergence capability and stability. The full-space adjoint, by correctly incorporating the sensitivity from backward-propagating cavity modes, provides a physically accurate gradient that guides the optimization robustly through the final, non-linear stages of the design process. This comparative result quantitatively confirms that for enclosed cavities where coherent multi-scattering is intrinsic, a full-space adjoint formulation is essential for obtaining high-fidelity solutions.

Supplementary Note 9. Convergence analysis for different patterns

Figure S9 shows the evolution of the AER over 120 optimization iterations for the patterns presented in Fig. 5A. The AER for every pattern drops rapidly within the first 40 - 60 iterations, followed by a period of finetuning and stabilization, a trend consistent with that observed for the E pattern (Fig. 3C). Specifically, the AER of the pentagram (image 9) improves from 2.5% to 0.3%, and that of the circular ring (image 10) improves from 4% to 0.9%. This consistent convergence behavior across geometrically diverse patterns confirms that our method is a general and practical design tool for shaping complex intracavity fields.

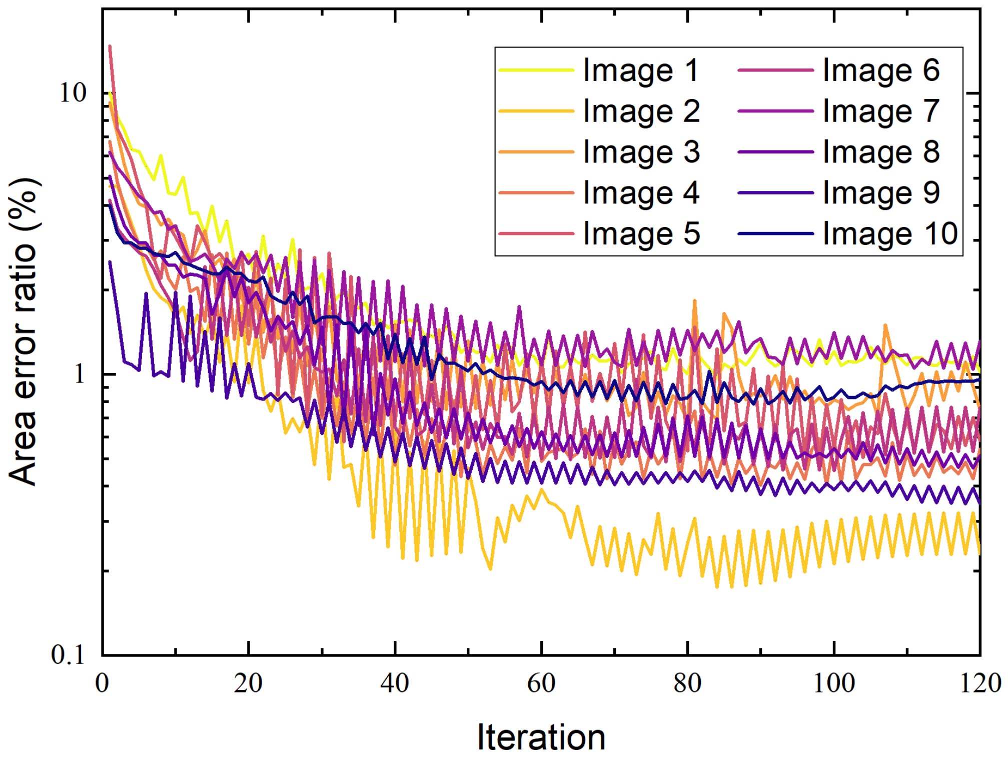

Figure S9 | Convergence of AER for different geometric patterns.

Supplementary Note 10. Simulation and experimental results of resolution test chart

As shown in Fig. S10, a typical non-periodic imaging pattern of resolution test chart with size of 5 μm $\times$ 3.5 μm is both numerically and experimentally demonstrated. In all the images, individual line features above 70 nm can be clearly distinguished, thus allowing us to determine the smallest feature width of $\sim$70 nm in the photoresist pattern (lines with a width of 70 nm and a center-to-center distance of 70 nm). In contrast to the widened imaging patterns before optimization, the distortion of the line widening can effectively eliminated by locally narrowing the metasurface mask, leading to a improvement of optimized patterns.

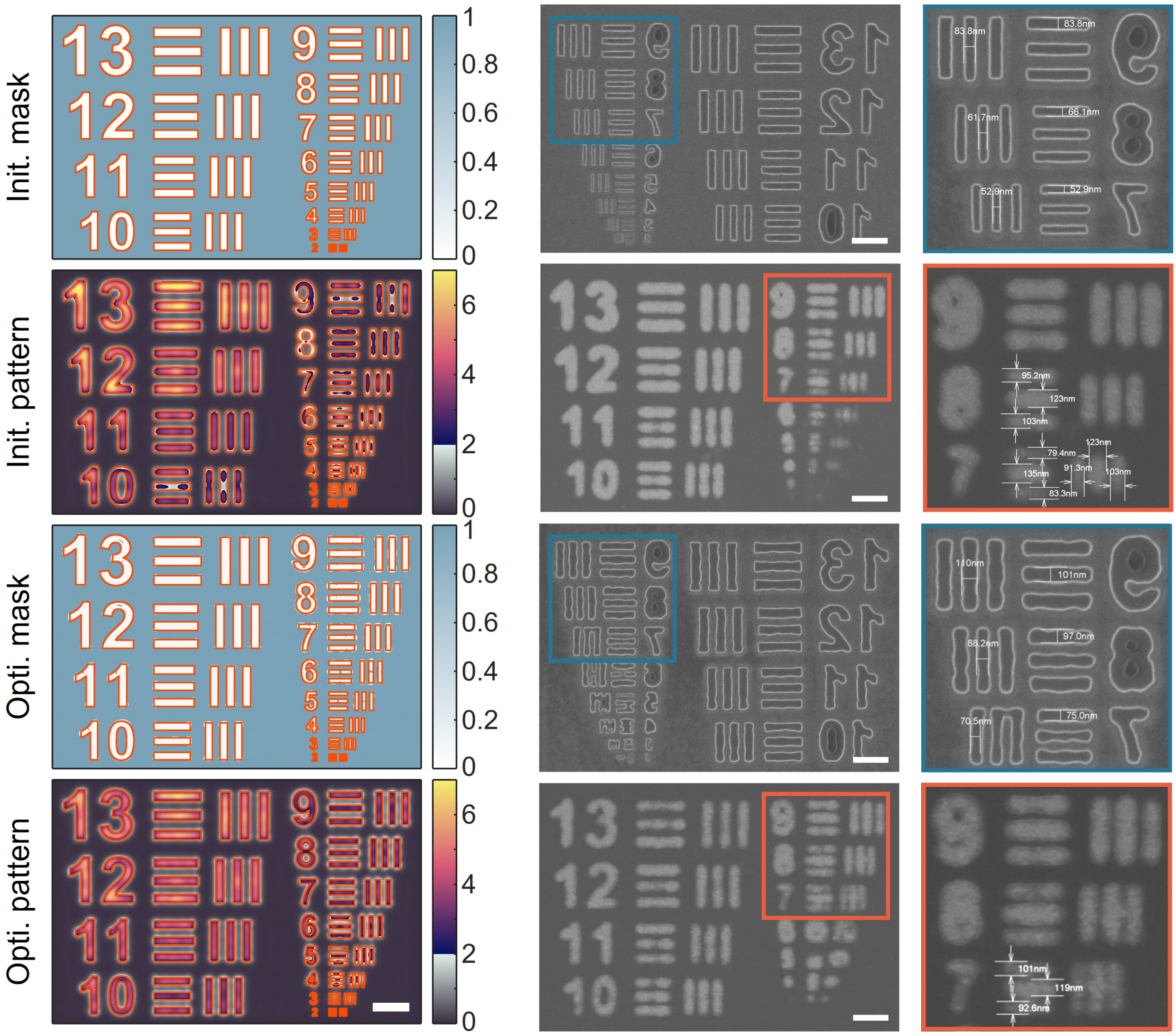

Figure S10 | Simulation and experiment results of resolution test chart for the initial and optimized metasurface masks and imaging pattern. The complete standard pattern consists of a total of 12 target elements with feature sizes ranging from 20 nm to 130 nm. The image size is 5 μm $\times$ 3.5 μm. Note that the mask patterns are inverted in the x-direction to compensate for the image mirroring effect inherent to the reflective plasmonic imaging system. Scale bar: 500 nm.

Supplementary Note 11. Simulation and experimental results of periodic patterns

Figure S11 indicates the experimental results of a periodic subwavelength array consisting of a rectangular bar and a closed ring. It is shown that the rectangular bars are optimized to be more curved compared to the initial metasurface mask, exhibiting as a “Y” structure on the end of the rectangles. It enables to suppress the overexposure of the rectangular bars and makes the imaging result more closer to the FoM. However, since the gap between the rectangular bars is only 50 nm beyond the optimization capability of presented SPP near-field imaging system, the actual imaging cannot achieve the resolution of ultra-high diffraction.

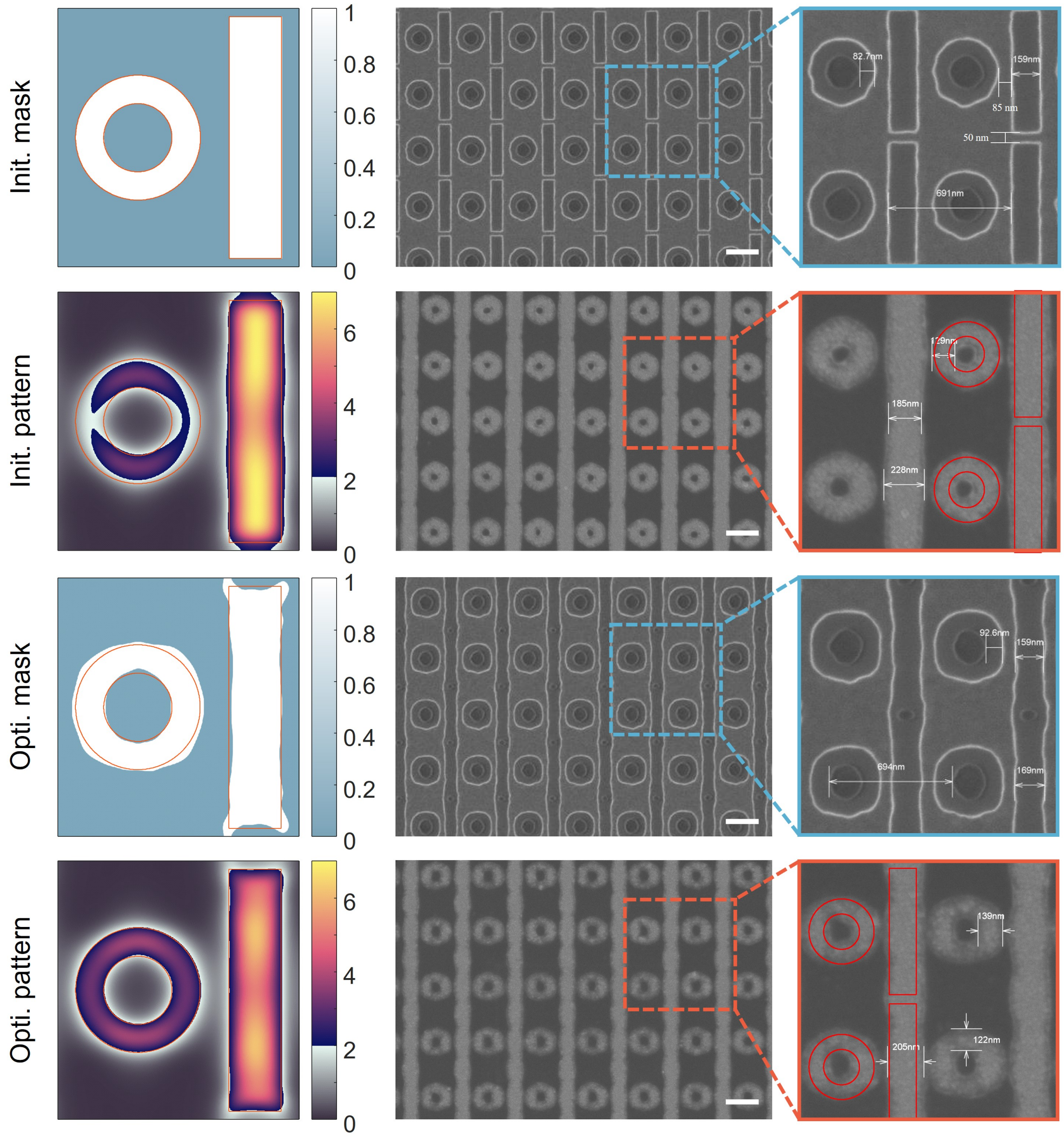

Figure S11 | Simulation and experiment results of periodic array for the initial and optimized metasurface masks and imaging pattern. Scale bar: 500 nm.

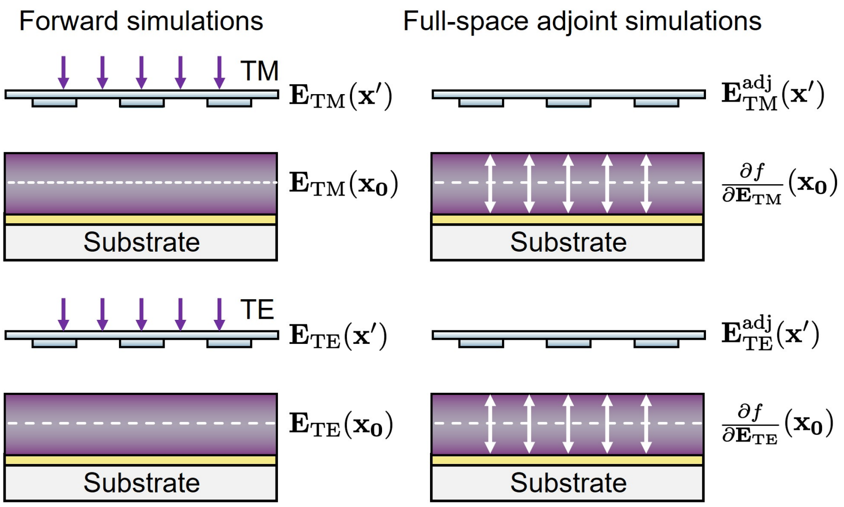

Figure S12 | Schematic diagram of forward and full-space adjoint simulation process under the polarization-dependent illumination in the SPP near-field imaging system.

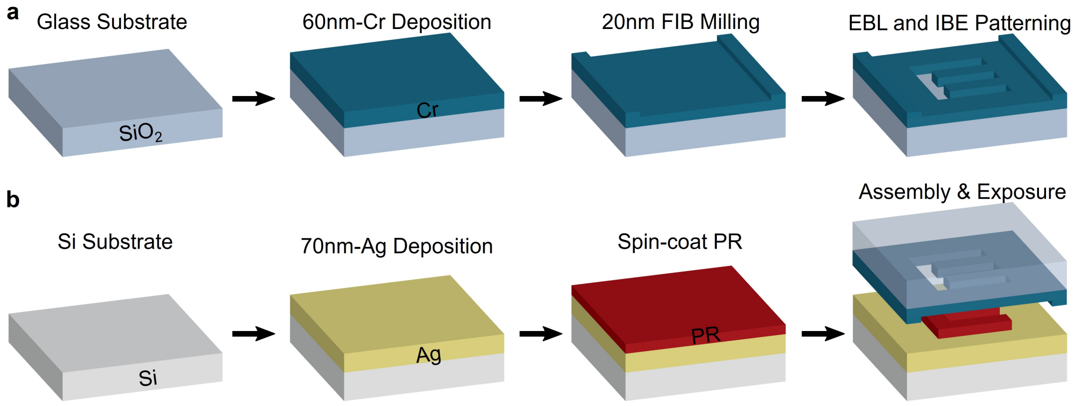

Figure S13 | Fabrication procedure of (a) Cr metasurface mask and (b) photoresist (PR) substrate for SPP near-field imaging.

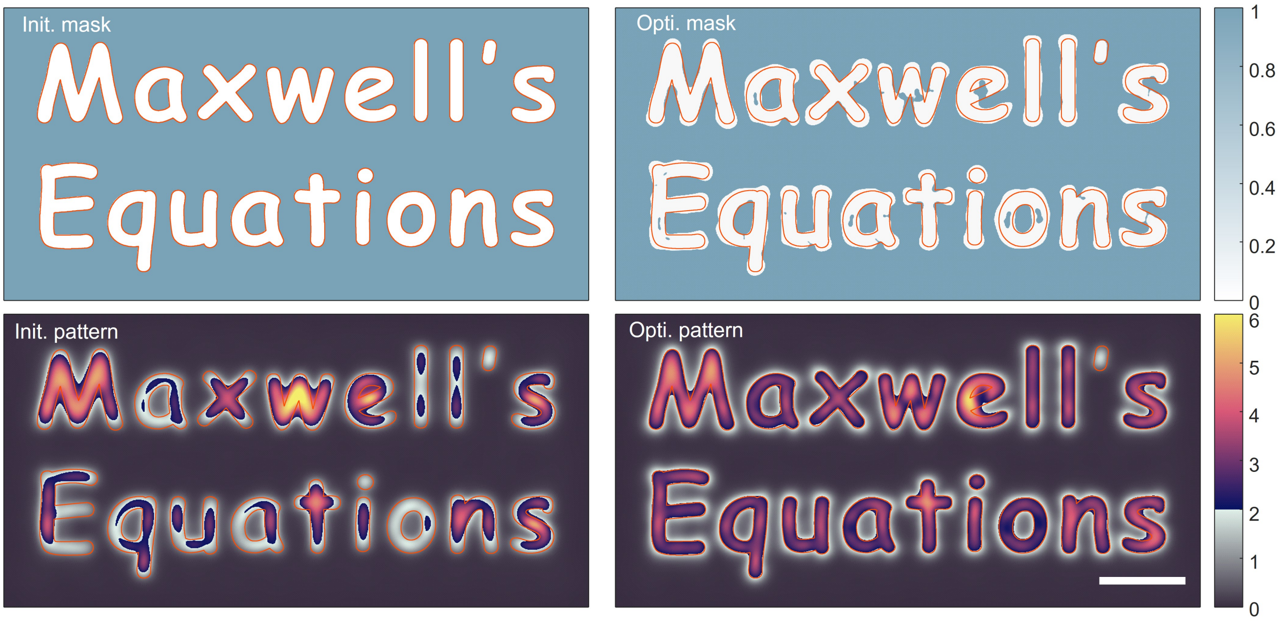

Figure S14 | Simulation results of large-area "Maxwell's Equation" image.
